# Functional traits of the world’s late Quaternary large-bodied avian and mammalian herbivores

**DOI:** 10.1038/s41597-020-00788-5

**Published:** 2021-01-20

**Authors:** Erick J. Lundgren, Simon D. Schowanek, John Rowan, Owen Middleton, Rasmus Ø. Pedersen, Arian D. Wallach, Daniel Ramp, Matt Davis, Christopher J. Sandom, Jens-Christian Svenning

**Affiliations:** 1 Centre for Compassionate Conservation, School of Life Sciences, University of Technology Sydney, Ultimo, Australia; 2 Center for Biodiversity Dynamics in a Changing World (BIOCHANGE), Department of Biology, Aarhus University, Aarhus, Denmark; 3 Section for Ecoinformatics and Biodiversity, Department of Biology, Aarhus University, Aarhus, Denmark; 4 Department of Anthropology, University at Albany, Albany, NY 12222 USA; 5 School of Life Sciences, University of Sussex, Sussex, UK; 6 Natural History Museum of Los Angeles County, Los Angeles, CA 90007 USA

**Keywords:** Palaeoecology, Conservation biology, Biodiversity

## Abstract

Prehistoric and recent extinctions of large-bodied terrestrial herbivores had significant and lasting impacts on Earth’s ecosystems due to the loss of their distinct trait combinations. The world’s surviving large-bodied avian and mammalian herbivores remain among the most threatened taxa. As such, a greater understanding of the ecological impacts of large herbivore losses is increasingly important. However, comprehensive and ecologically-relevant trait datasets for extinct and extant herbivores are lacking. Here, we present *HerbiTraits*, a comprehensive functional trait dataset for all late Quaternary terrestrial avian and mammalian herbivores ≥10 kg (545 species). *HerbiTraits* includes key traits that influence how herbivores interact with ecosystems, namely body mass, diet, fermentation type, habitat use, and limb morphology. Trait data were compiled from 557 sources and comprise the best available knowledge on late Quaternary large-bodied herbivores. *HerbiTraits* provides a tool for the analysis of herbivore functional diversity both past and present and its effects on Earth’s ecosystems.

## Abstract (summary)

**Table:** 

Measurement(s)	body weight • diet • digestion trait • habitat • limb morphology trait
Technology Type(s)	digital curation
Factor Type(s)	species of avian and mammalian herbivores
Sample Characteristic - Organism	avian herbivores • mammalian herbivores
Sample Characteristic - Environment	terrestrial biome

Machine-accessible metadata file describing the reported data: 10.6084/m9.figshare.13353416

## Background & Summary

Large-bodied terrestrial avian and mammalian herbivores strongly influenced terrestrial ecosystems through much of the Cenozoic–the last 66 million years of Earth history. However, many of the world’s large-bodied herbivore species became extinct or experienced significant range contractions beginning ~100,000 years ago in the late Quaternary. Human impacts were the primary driver of these extinctions and declines, though possibly in conjunction with climate change^1–3^. The world’s remaining large-bodied herbivores are among the most threatened species on the planet^4,5^, leading to urgent calls to protect these species and to better understand their distinct ecological roles^6^.

Large-bodied herbivores are unique in their capacity to consume large quantities of plant biomass and, as the largest terrestrial animals, they are uniquely capable of causing disturbance to vegetation and soils. These taxa thus exert strong top-down control on ecological communities and ecosystem processes. Prehistoric and historic losses of large herbivores led to profound changes to Earth’s terrestrial ecosystems, including reductions in ecosystem productivity from reduced nutrient cycling, reduced carbon forest stocks from the loss of disturbance, increases in wildfire frequency and severity, and changes in plant communities^7–12^. The causes and ecological legacies of late Quaternary extinctions are key topics of rapidly growing research interest^13–18^. Likewise, the potential for introduced herbivores (either inadvertently or intentionally) to restore lost ecological processes is an important focus of research and debate today^19–27^.

**Fig. 1:**
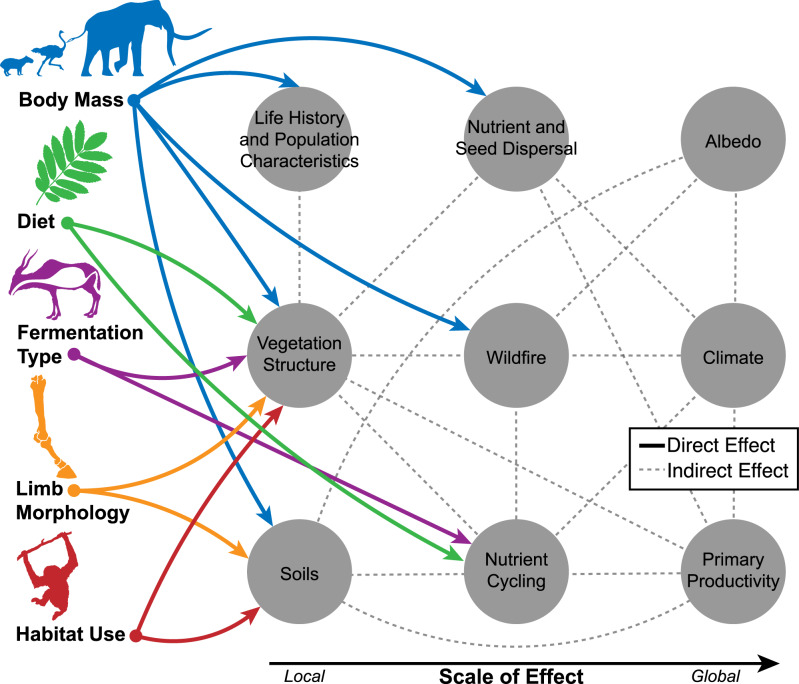
Herbivores affect numerous ecological and ecosystem processes. The traits contained in *HerbiTraits* encapsulate major dimensions of herbivore ecology and its effect on the environment, from affecting local vegetation and soils to influencing global climate. Linkages indicate direct and indirect effects of traits on ecological processes or components, scaling from traits (left-hand side) to globe (right-hand side).

The capacity for organisms to affect the environment is driven by their functional trait combinations^28^ (Fig. 1). As such, the availability and accuracy of herbivore functional trait data is critical for understanding the patterns and ecological consequences of the late Quaternary extinctions, the implications of modern ecological changes, and to guide conservation action. However, datasets of herbivore traits are rare and suffer from poor documentation, incomplete species lists, and outdated taxonomies. Trait datasets have been particularly scarce and/or inconsistently available for extinct species. Furthermore, there often exists a trade-off between species coverage and the resolution of many datasets. Mammalian trait datasets such as PHYLACINE^29^ or MOM (Mass of Mammals)^30^ include data on many late Quaternary mammal species, including carnivorous, aquatic, and flying species. These datasets thus include traits that are universal across these disparate ecological niches but in doing so lack trait data relevant to understanding herbivores and their unique ecological roles in particular. Furthermore, few datasets have considered or included avian herbivores, which can be particularly important components of large vertebrate faunas, especially on islands. The lack of a consistent and high-resolution trait dataset for late Quaternary avian and mammalian herbivores stymies efforts to understand the consequences of ecological changes that followed late Quaternary extinctions and hinders modern responses to changes in this important functional group.

Here, we present *HerbiTraits*, a comprehensive global trait dataset containing functional traits for all terrestrial avian (*n* = 34 species) and mammalian (511 species) herbivores ≥10 kg spanning the last ~130,000 years of the late Quaternary. *HerbiTraits* contains traits fundamental to understanding the multiple dimensions of herbivore ecology, including body mass, diet, fermentation type, habitat use, and limb morphology (Fig. 1, Table 1). These data are broadly useful for both paleo and modern ecological research, including potential conservation and rewilding efforts involving herbivores. Recent research using these data has yielded insight into the functionality of novel assemblages composed of introduced and native herbivores^25^.

**Table 1:** *HerbiTraits* contains key traits for all late Quaternary herbivorous mammals over the last 130,000 years.

Trait	Variable Name	Variable type	Values/Unit	Notes
Mass	Mass.g	Continuous	Grams	Body mass is a continuous variable reflecting average body mass of adult, across males and females.
Diet	Three variables: *Diet.Graminoids;* *Diet.Browse.Fruit;* *Diet.Meat*	Ordinal	0 (insignificant, 0–9% of diet) 1 (low significance, 10–24%) 2 (moderate significance, 25–49%) 3 (high significance, 50–100%)	Graminoid, browse, and meat consumption were treated as separate ordinal variables. Fruit consumption was included with browse. Grass-seed, bamboo, and forbs (herbaceous dicots) were considered browse^39^.
Fermentation type	Two variables: *Fermentation.Type* *Fermentation.Efficiency*	Categorical/Ordinal	Simple gut (Efficiency: 0) Hindgut colon (Efficiency: 1) Hindgut caecum (Efficiency: 1) Foregut non-ruminant (Efficiency: 2) Foregut ruminant (Efficiency: 3)	Fermentation type was collected as a categorical variable, following^46^, but was ranked as an ordinal variable in terms of efficiency (0–3).
Habitat	Three variables: *Habitat.Aquatic;* *Habitat.Terrestrial* *Habitat.Arboreal*	Binary	0 (no significant use of habitat) 1 (use of habitat)	Use of particular habitats (aquatic, terrestrial, arboreal) was given a 0 or 1, non-exclusively. This variable has also been coded categorically and includes *semi-aquatic, terrestrial, semi-arboreal, arboreal*
Limb morphology	*Limb.Morphology*	Categorical	Plantigrade Digitigrade Unguligrade	Limb morphology reflects major vertebrate postural adaptations, which govern habitat affinities, fossoriality, cursoriality, and disturbance-related effects on soils.

Traits include body mass, diet, fermentation type, habitat, and limb morphology. These variables can be used to understand patterns and processes of ecological change.

## Methods

### Compilation of Species List

*HerbiTraits* includes all known herbivores over the last ~130,000 years from the start of the last interglacial period, which is ~30,000 years prior to onset of the earliest late Quaternary extinctions. The mammal species list was derived from PHYLACINE v1.2.1^29^. Herbivorous birds ≥10 kg were gathered through a comprehensive review of the peer-reviewed literature, including handbooks^31^. Herbivores were selected as any species ≥10 kg with >50% plant in their diet, thus including several omnivorous taxa (e.g. bears). The 10 kg cut-off was chosen following Owen-Smith’s^32^ designation of a mesoherbivore, a category paradigmatic to many herbivore ecological analyses^33^ but missed by the commonly used ≥44 kg cutoff commonly used for ‘megafauna’^34^. Domestic species with wild introduced populations (e.g. horses *Equus ferus caballus*, water buffalo *Bubalus arnee bubalis*)^26^ were included separately in *HerbiTraits* as their trait values (particularly body mass) can differ substantially from their extant or extinct pre-domestic conspecifics. We included the status for all species, including ‘Extant’, ‘Extinct before 1500 CE’, ‘Extinct after 1500 CE’, ‘Extinct before 1500 CE, but wild in introduced range’ and ‘Extinct after 1500 CE, but wild in introduced range’. The latter two cases apply to species that are extinct in their native ranges (e.g. *Camelus dromedarius*, *Bos primigenius, Oryx dammah*) but which have wild, introduced populations. Species listed as Extinct in the Wild by the IUCN Red List are considered ‘Extinct after 1500 CE’ in the dataset.

### Functional Traits

Functional trait data were collected from a variety of peer-reviewed literature (n = 502 references, 91% of total references), books (n = 28, e.g. *Handbook of the Mammals of the World*^35^), online databases (*n* = 7), theses (*n* = 9), and others (*n* = 11). For all taxa, multiple sources were consulted, and the most reliable source was used in trait designation. Reliability was based on the method of the source data (see Table 2 for the ranking system we employed). In cases where studies disagreed, we gave extra weight to studies with more reliable methods, larger sample sizes, and/or broader geographic and temporal coverage. We provide justification for our decision-making process in note fields.

**Table 2:** Method for assigning reliability in trait assessments for all traits.

Reliability rank	Diet	Mass	Habitat	Fermentation & Limb morphology
0	Expert opinion Imputed Inferred from ___: *Genus, habitat associations*	Expert Opinion Imputed Method unknown Method uncertain Inferred from ___: *relative of suggested similar size* *Extant species (method unknown, presume measured)*	Expert opinion Imputed Inferred from ___: *Family, Genus, absence of evidence, co-occurring species*	Proxy isotopes *____*: (*δ*^13^*C, δ*^18^*O, δ*^15^*N*) Proxy ___ morphology: *craniodental, appendicular* Inferred from ____: *diet, Class, Infraclass, Order*
1	Functional ___ morphology: *craniodental, appendicular* Observed low sample size (1–5) ___: *dental bolus/coprolite*	Functional isometric relationship (____): *bone size, shoulder height, teeth size etc*.	Functional ____ morphology: *craniodental, appendicular* Observed low (1–5) sample size ___: *dental bolus, coprolite, foraging, fecal analysis, stomach-contents, DNA metabarcoding*	Inferred from ____: *Suborder, sister Families*
2	Proxy ___: *isotopes (δ*^13^*C, δ*^18^*O, δ*^15^*N, etc), dental wear (mesowear, microwear)*	Proxy allometric relationship (*___*)*: bone size, shoulder height, teeth size etc*, Proxy volumetric estimate	Inferred from ___: *habitat association, vegetation association*	Inferred from ___: *Subfamily, Family*
3	Observed ___: *Fecal analysis, stomach-contents, coprolites, foraging observations, dental bolus, DNA metabarcoding*	Observed measured body mass	Observed ___: *foraging observations, habitat use*	Observed ___: *Species, Genus*

The italic text substitutes for ‘___’. At times, in cases where sources contradicted each other, or because of low source quality, reliability rank will be lower than reported here, with explanations in respective notes column. Likewise, particularly for diet, if empirical evidence was interpolated from a closely related species, the taxonomic relation is noted in parentheses following the method designation and reliability ranks are reduced.

#### Body mass

Body mass is strongly associated with a number of life history attributes and ecological effects, including metabolic and reproductive rates, the capacity to cause disturbance, the ability to digest coarse fibrous vegetation, and the vulnerability of herbivores to predation^32,36^ (Fig. 1). Mammal body mass (in grams) was sourced from PHYLACINE v1.2.1^29^ and Mass of Mammals^30^(Table 1). Avian body masses were collected directly from the literature. We collected body mass data separately for domesticated species from AnAge: Animal Senescence and Aging database^37^, because their body masses can vary drastically from their pre-domesticated relatives.

Given variability in mass estimation methods and their reliability, we tracked down the primary sources that the aforementioned datasets cited and coded the mass estimation method used. In general, the most reliable body mass estimates for extinct mammals were calculated with volumetric estimates (e.g. by measuring displacement of a fluid) or by allometric scaling equations. Isometric equations (which assume a simple linear relationship between morphology (e.g. tarsus length) and body mass were ranked lower, as were cases where body masses were estimated based on similar, often closely related species (Table 2). However, we restricted metadata gathering to extinct taxa as accounts of extant species rarely report how their mass estimates were generated (though in all likelihood they are derived from a measured voucher specimen). Furthermore, the mass estimates of extinct species are the most uncertain and the most difficult to verify for users who are not familiar with extinct species or paleobiological methods of mass reconstruction. The avian mass estimates were collected by the authors directly from the peer reviewed literature.

#### Diet

Diet determines the type of plants herbivores consume and thus downstream effects on vegetation, nutrient cycling, wildfire, seed dispersal, and albedo (Fig. 1)^19,33^. Diet was collected as three ordinal variables describing graminoid consumption (i.e. grazing), browse and fruit consumption (i.e. browsing), and meat consumption (including vertebrate and invertebrate) (Table 1). Grazing and browsing have distinct effects on vegetation and ecosystems and are key dimensions of herbivore dietary differentiation^33^, reflecting a suite of strategies that have evolved across all major herbivore lineages. This is because grasses and their relatives (graminoids) and dicots (woody plants and herbaceous forbs) present different obstacles to herbivory. While graminoids are highly abrasive and composed primarily of cellulose, dicots are lignified and/or protected with secondary chemical compounds^38^. Frugivory is often impossible to differentiate from browsing based on paleobiological sources of data for extinct taxa and thus was included with browsing, though known records of fruit consumption are marked in the dataset’s diet notes column. The consumption of bamboo was considered browsing despite bamboo being a grass, as its lignification makes it structurally similar to wood^39^.

Graminoid, browse, and meat consumption ranged from 0–3, with 0 indicating insignificant consumption and 3 indicating regular or heavy consumption. In general, 0 indicates 0–9% of diet, 1 indicates 10–19%, 2 indicates 20–49%, and 3 indicates 50–100%. For example, an obligate grazer that consumes 90% graminoids would have a 0 for browse, and a 3 for graze, whereas a grazer that consumes 70% graze and 30% browse would have a 3 for graze and a 2 for browse. Likewise, if a species consumed both graze and browse equally (e.g. a mixed feeder) they would receive a score of 3 for each. While dietary estimates for extinct taxa by necessity came from broad temporal and spatial scales^40^, the coarseness of our ordinal (0–3) diet designation allowed us to capture intraspecific and spatiotemporal variation, making extant and extinct species comparable.

Diets for extant species (n = 321) were based on records from the *Handbook of the Mammals of the World*^35^, which represents a compiled, expert-reviewed synopsis of natural history data across mammals. However, to ensure that these diet designations were up to date, we conducted literature reviews for each species, searching for any papers published since the *Handbook of the Mammals of the World* (2009–2011 depending on taxonomic group). We also consulted region-specific handbooks, in particular Kingdon *et al*. 2013 *Mammals of Africa*^41^. In cases where percent diet composition was unavailable, we determined dietary values by converting textual descriptions into ordinal values (Table 3) following the methods outlined by MammalDIET^42^. Diets for extinct species were gathered from a variety of literature, as no systematic compilation of extinct herbivore diet is presently available. Discrepancies between sources were noted and described in the dietary notes field.

**Table 3:** Method for converting textual descriptions to ordinal dietary values.

Diet Value	Interpretation	Textual description
3	The food source is a major (51–100%) and essential part of the species’ diet.	*“primarily consumes”* *“mainly consumes”* *“regularly consumes”* *“major part of the diet”* *“only consumes”*
2	The food source is an important but not major part (21–50%) of the species diet. It is generally a non-essential part of the species diet.	*“also consumes”* *“seasonally consumes”* *“may consume”*
1	The food source is a relatively small (11–20%) and unimportant part of the species diet.	*“occasionally consumes”* *“sometimes consumes”* *“opportunistically consumes”* *“has been reported to eat”*
0	This food source is an insignificant part (0–10%) of the species diet.	*“does not consume”* *“has once been seen consuming”* The text does not mention the food source

This table is based upon the method outlined by Kissling *et al*.^42^, and shows some example key words and phrases that were used to determine dietary values.

The methods of the original source papers for extant and extinct were designated and ranked by reliability (Table 2), which was used in assigning final dietary values. We gave priority to direct observations, including fecal or stomach content analysis, coprolites, fossilized boluses (e.g. phytoliths or other vegetation remnants in teeth), and foraging observations. This category was followed by proxy data, such as stable carbon isotopes and dental microwear and mesowear. Inferences from functional morphology, direct observations with sample sizes ≤5, expert opinions, and inferences from extant relatives were considered to have the lowest reliability (Table 2).

Herbivore diets can be highly variable, particularly across seasons and regions. In most cases where primary sources differed because of geographic variation in diets (e.g. a diet heavy in grass in one location and in browse in another), we increased the value of both dietary categories to reflect the mixed feeding capacity of the species across their range. However, we tempered this in cases of unusual diets in response to starvation, such as in the case of severe droughts, as consumption does not necessarily mean the species has the capacity to survive on these alternative diets. In these cases, we have noted the evidence and justified our decision-making process.

In cases where no dietary data were available (*n* = 26 species), we imputed diet values based on a posterior distribution of 1,000 equally-likely phylogenies for mammals ≥10 kg from PHYLACINE v1.2.1^29,43^. We used the R package “Rphylopars” v0.3.0 with a Brownian motion evolutionary model and took the median value from the 1,000 phylogenetic trees^44,45^. This model accounted for both the evolutionary correlation of the individual dietary values across the full phylogeny as well as the probability of diet values based on other traits, as some trait combinations (e.g. arboreality and grazing) are very rare. Given that this imputation was conducted across full mammal phylogenies (≥10 kg), we used life history traits from PHYLACINE v1.2.1^29,43^, so that imputation for species only distantly related to other herbivores (e.g. bears) would be robust.

Ordinal diet scores were further used to categorize species into two types of dietary guild classifications, one herbivore-specific which contained browsers (graze = 0-1, browse = 3), mixed-feeders (graze = 2-3, browse = 2-3), and grazers (graze = 3, browse = 0-1), and another guild containing omnivores (any species with meat consumption ≥2). Users can easily derive finer-scale dietary guilds (e.g. mixed-feeder preferring browse) from the ordinal scores if desired.

#### Fermentation type

Digestive physiology controls the quantity and quality of vegetation (e.g., fiber and nutrient content) that herbivores consume. Fermentation type therefore shapes effects on vegetation, gut passage rate, seed and nutrient dispersal distances, water requirements, and the resulting stoichiometry of excreta^19,46–49^ (Fig. 1). Following Hume^46^, fermentation type was collected as a categorical variable consisting of simple gut, hindgut colon, hindgut caecum, foregut non-ruminant, and ruminant (Table 1). These variables capture the range of fermentation adaptations across avian and mammalian herbivores. Based on these classifications and Hume^46^, we also ranked fermentation efficiencies (0–3) on an ordinal scale to these various digestive strategies, to facilitate quantitative functional diversity analyses (Table 1).

Fermentation types show strong phylogenetic conservatism at the family level. Therefore, for the most part, if direct anatomical evidence was not available, we inferred fermentation types from extant relatives. However, some extinct herbivores possess no close modern relatives and may have been functionally non-analog (e.g. 23 extinct ground sloths, 3 notoungulates, 4 diprotodons, 16 glyptodonts, and 12 giant lemurs). In these cases, closest living relatives, expert opinions, and craniodental morphology were used to determine the most likely fermentation system. For example, notoungulates, an extinct group from South America, possess no close relatives yet their craniodental and appendicular morphology resemble extant hindgut fermenting taxa (rhinos), and hindgut fermentation is widely considered to be ancestral in ungulates^50^. In all cases, we describe our justification and the state of the debate in the current literature.

#### Habitat use

Habitat use determines the components of ecosystems that herbivores interact with and is central to understanding their effects on vegetation, soils, and processes like nutrient dispersal (e.g. moving nutrients from terrestrial to aquatic environments^51^). We classified habitat with three non-exclusive binary variables (0 or 1) for the use of arboreal, terrestrial, and aquatic environments. We further classified this variable categorically as semi-aquatic, terrestrial, semi-arboreal, and arboreal. Defining habitat use is challenging as many terrestrial species use aquatic or arboreal environments opportunistically, and percentage habitat use data is unavailable for most species. To ensure habitat designations were consistent for extant and extinct species, we classified taxa on the basis of obligate habitat use across their geographic range and/or the possession of specialized adaptations (e.g. climbing ability) that would be evident in the morphology of fossil specimens. Further proof of habitat use by extinct species was inferred from close relatives or isotopic proxy data, when relevant. In cases where no specific information was available, we inferred habitat use from absence of evidence (e.g. there is no specific data regarding aquatic or arboreal habitat use by gemsbok *Oryx gazella*).

#### Limb morphology

Limb morphology is broadly associated with herbivore habitat preferences, locomotion (e.g., cursoriality, fossoriality, climbing), anti-predator responses, and rates of body size evolution^52–54^. Limb morphology also controls disturbance-related trampling effects on soils, with hoofed unguligrade taxa having stronger influences on soils than those with other morphologies^55^. Trampling has important effects on soils, hydrology, albedo, and vegetation^7,56^ and is often considered an essentially novel aspect of introduced herbivores in Australia and North America (e.g^10,57,58^.). Limb morphology was collected as a three-level categorical variable consisting of plantigrade (walking on soles of feet), digitigrade (walking on toes), and unguligrade (walking on hoof). For example, plantigrade species are more likely to be fossorial or scansorial in habit, digitigrade species are likely to be saltatory or ambulatory (e.g. extant kangaroos), while unguligrade species are often adapted for rocky, vertiginous terrain or cursoriality^53,54^. Limb morphology shows high phylogenetic conservatism across herbivore lineages and thus was primarily collected at the genus or family level from primary and secondary literature.

## Data Records

*HerbiTraits* consists of an Excel workbook containing metadata (column names and descriptions), the trait dataset, and references as three separate sheets. The dataset is open-access and is hosted on Figshare^59^ as well as on GitHub (https://github.com/MegaPast2Future/HerbiTraits).

## Technical Validation

The majority of functional trait data were collected from primary peer-reviewed literature (1,733 trait values from 456 articles), secondary peer-reviewed literature (1,294 values from 46 articles), or academic handbooks (1,099 trait values from 27 resources). Twenty-eight remaining resources consisted of theses (*n* = 39 trait values), databases (44), websites (39), conference proceedings (9), and grey literature (5). For transparency, justifications for trait designations (particularly relevant for extinct species) are described in the Notes columns and the highest quality evidence is ranked in trait-specific Reliability columns. Contradictions between sources have been noted and values have been based on the most empirically-robust methods or by averaging values across studies (see above). All data designations have been cross-checked (by EJL, SDS, JR, MD, and OM). We aim to maintain *HerbiTraits* with the best available data. We urge users to report errors or updates on newly published data for integration into *HerbiTraits* by filing an Issue on our GitHub (https://github.com/MegaPast2Future/HerbiTraits) repository page, or by emailing the corresponding authors. Furthermore, the GitHub (https://github.com/MegaPast2Future/HerbiTraits) page includes an incomplete trait file, which contains other ecologically relevant traits, such as adaptations for digging and free water dependence^60^. These traits remain unavailable for many taxa, but provide a starting point for further data collection and analysis.

## Usage notes

Please cite this publication when using *HerbiTraits*. As the taxonomy and phylogeny is derived from PHYLACINE v1.2.1, that data is compatible with PHYLACINE v1.2.1’s phylogeny and range maps and with the IUCN Red List Version 2016-3 (2016), with the exception of domestic mammals and birds. All source references are cited in the main text^14,29,61–601^. Where possible, we have coded trait data in duplicate ways to facilitate different types of analysis. For example, diet, fermentation, and habitat use, are coded both as categorical variables and as ordinal/binary variables for use in functional diversity analyses.

## Data Availability

The authors declare no custom code necessary for the interpretation or use of dataset.

## References

[1] Barnosky AD, Koch PL, Feranec RS, Wing SL, Shabel AB (2004). Assessing the causes of late Pleistocene extinctions on the continents. Science.

[2] Sandom C, Faurby S, Sandel B, Svenning JC (2014). Global late Quaternary megafauna extinctions linked to humans, not climate change. Proc. R. Soc. B..

[3] Metcalf JL (2016). Synergistic roles of climate warming and human occupation in Patagonian megafaunal extinctions during the Last Deglaciation. Science Advances.

[4] Ripple WJ (2015). Collapse of the world’s largest herbivores. Science Advances.

[5] Atwood TB (2020). Herbivores at the highest risk of extinction among mammals, birds, and reptiles. Science Advances.

[6] Ripple WJ (2016). Saving the world’s terrestrial megafauna. Bioscience.

[7] Zimov SA (1995). Steppe-tundra transition: a herbivore-driven biome shift at the end of the Pleistocene. The American Naturalist.

[8] Zhu D (2018). The large mean body size of mammalian herbivores explains the productivity paradox during the Last Glacial Maximum. Nature Ecology & Evolution.

[9] Berzaghi F (2019). Carbon stocks in central African forests enhanced by elephant disturbance. Nature Geoscience.

[10] Johnson, C. N. *et al*. Can trophic rewilding reduce the impact of fire in a more flammable world? *Philos. Trans. R. Soc. Lond. B Biol. Sci*. **373**, 10.1098/rstb.2017.0443 (2018). 10.1098/rstb.2017.0443 PMC6231065 30348870

[11] Rule S (2012). The aftermath of megafaunal extinction: ecosystem transformation in Pleistocene Australia. Science.

[12] Gill JL, Williams JW, Jackson ST, Lininger KB, Robinson GS (2009). Pleistocene megafaunal collapse, novel plant communities, and enhanced fire regimes in North America. Science.

[13] Smith FA, Elliott Smith RE, Lyons SK, Payne JL (2018). Body size downgrading of mammals over the late Quaternary. Science.

[14] Smith FA (2015). Unraveling the consequences of the terminal Pleistocene megafauna extinction on mammal community assembly. Ecography.

[15] Davis M (2017). What North America’s skeleton crew of megafauna tells us about community disassembly. Proc. R. Soc. B..

[16] Bakker ES (2016). Combining paleo-data and modern exclosure experiments to assess the impact of megafauna extinctions on woody vegetation. Proc. Natl. Acad. Sci. USA.

[17] Bakker ES, Arthur R, Alcoverro T (2016). Assessing the role of large herbivores in the structuring and functioning of freshwater and marine angiosperm ecosystems. Ecography.

[18] Rowan, J. & Faith, J. in *The Ecology of Browsing and Grazing II* 61–79 (Springer, 2019).

[19] Wallach AD (2018). Invisible megafauna. Conservation Biology..

[20] Sandom CJ (2020). Trophic rewilding presents regionally specific opportunities for mitigating climate change. Philosophical Transactions of the Royal Society B.

[21] Svenning JC (2016). Science for a wilder Anthropocene: Synthesis and future directions for trophic rewilding research. Proc. Natl. Acad. Sci. USA.

[22] Guyton, J. A. *et al*. Trophic rewilding revives biotic resistance to shrub invasion. *Nature Ecology & Evolution*, 10.1038/s41559-019-1068-y (2020). 10.1038/s41559-019-1068-y 31932702

[23] Derham TT, Duncan RP, Johnson CN, Jones ME (2018). Hope and caution: rewilding to mitigate the impacts of biological invasions. Philos. Trans. R. Soc. Lond. B Biol. Sci..

[24] Derham T, Mathews F (2020). Elephants as refugees. People and Nature.

[25] Lundgren, E. J. *et al*. Introduced herbivores restore Late Pleistocene ecological functions. *Proc. Natl. Acad. Sci. USA*, 10.1073/pnas.1915769117 (2020). 10.1073/pnas.1915769117 PMC7148574 32205427

[26] Lundgren EJ, Ramp D, Ripple WJ, Wallach AD (2018). Introduced megafauna are rewilding the Anthropocene. Ecography.

[27] Donlan CJ (2006). Pleistocene rewilding: an optimistic agenda for twenty-first century conservation. The American Naturalist.

[28] Luck GW, Lavorel S, McIntyre S, Lumb K (2012). Improving the application of vertebrate trait-based frameworks to the study of ecosystem services. J. Anim. Ecol..

[29] Faurby S (2018). PHYLACINE 1.2: The Phylogenetic Atlas of Mammal Macroecology. Ecology.

[30] Smith FA (2003). Body mass of late Quaternary mammals. Ecology.

[31] Hume, J. P. & Walters, M. *Extinct birds*. Vol. 217 (A&C Black, 2012).

[32] Owen-Smith, R. N. *Megaherbivores: the influence of very large body size on ecology*. (Cambridge University Press, 1988).

[33] Gordon, I. J. & Prins, H. H. *Ecology Browsing and Grazing II*. (Springer Nature, 2019).

[34] Martin, P. S. & Wright, H. E. *Pleistocene extinctions; the search for a cause*. (National Research Council (U.S.): International Association for Quaternary Research., 1967).

[35] Wilson, D. E. & Mittermeier, R. A. *Handbook of the Mammals of the World* Vol. 1-9 (Lynx Publishing, 2009-2019).

[36] Hopcraft JGC, Olff H, Sinclair ARE (2010). Herbivores, resources and risks: alternating regulation along primary environmental gradients in savannas. Trends Ecol. Evol..

[37] AnAge: The Animal Ageing and Longevity Database. (2020).

[38] Clauss, M., Kaiser, T. & Hummel, J. in *The ecology of browsing and grazing* 47-88 (Springer, 2008).

[39] Van Soest PJ (1996). Allometry and ecology of feeding behavior and digestive capacity in herbivores: a review. Zoo Biology: Published in affiliation with the American Zoo and Aquarium Association.

[40] Davis M, Pineda-Munoz S (2016). The temporal scale of diet and dietary proxies. Ecol. Evol..

[41] Kingdon, J. *et al*. *Mammals of Africa*. Vol. I-VI (Bloomsbury Natural History, 2013).

[42] Kissling WD (2014). Establishing macroecological trait datasets: digitalization, extrapolation, and validation of diet preferences in terrestrial mammals worldwide. Ecol. Evol..

[43] Faurby S, Svenning JC (2015). A species-level phylogeny of all extant and late Quaternary extinct mammals using a novel heuristic-hierarchical Bayesian approach. Mol. Phylogenet. Evol..

[44] Goolsby EW, Bruggeman J, Ané C (2017). Rphylopars: fast multivariate phylogenetic comparative methods for missing data and within‐species variation. Methods Ecol. Evol..

[45] Bruggeman J, Heringa J, Brandt BW (2009). PhyloPars: estimation of missing parameter values using phylogeny. Nucleic Acids Res..

[46] Hume ID (2002). Digestive strategies of mammals. Acta Zoologica Sinica.

[47] Demment MW, Van Soest PJ (1985). A nutritional explanation for body-size patterns of ruminant and nonruminant herbivores. The American Naturalist.

[48] Doughty CE (2016). Global nutrient transport in a world of giants. Proc. Natl. Acad. Sci. USA.

[49] Hofmann RR (1989). Evolutionary steps of ecophysiological adaptation and diversification of ruminants: a comparative view of their digestive system. Oecologia.

[50] Prothero, D. R. & Foss, S. E. *The evolution of artiodactyls*. (JHU Press, 2007).

[51] Subalusky AL, Dutton CL, Rosi-Marshall EJ, Post DM (2015). The hippopotamus conveyor belt: vectors of carbon and nutrients from terrestrial grasslands to aquatic systems in sub-Saharan Africa. Freshw. Biol..

[52] Kubo T, Sakamoto M, Meade A, Venditti C (2019). Transitions between foot postures are associated with elevated rates of body size evolution in mammals. Proc. Natl. Acad. Sci. USA.

[53] Brown JC, Yalden DW (1973). The description of mammals-2 limbs and locomotion of terrestrial mammals. Mammal Review.

[54] Polly, P. D. in *Fins in*to Li*mb*s: Ev*olution, Development, and Transformation* (ed B.K. Hall) 245-268 (2007).

[55] Cumming DHM, Cumming GS (2003). Ungulate community structure and ecological processes: body size, hoof area and trampling in African savannas. Oecologia.

[56] te Beest M, Sitters J, Ménard CB, Olofsson J (2016). Reindeer grazing increases summer albedo by reducing shrub abundance in Arctic tundra. Environmental Research Letters.

[57] Bennett M (1999). Foot areas, ground reaction forces and pressures beneath the feet of kangaroos, wallabies and rat-kangaroos (Marsupialia: Macropodoidea). J. Zool..

[58] Beever EA, Huso M, Pyke DA (2006). Multiscale responses of soil stability and invasive plants to removal of non‐native grazers from an arid conservation reserve. Diversity and Distributions.

[59] Lundgren EJ (2020). figshare.

[60] Kihwele ES (2020). Quantifying water requirements of African ungulates through a combination of functional traits. Ecological Monographs.

[61] Abbazzi L (2004). Remarks on the validity of the generic name *Praemegaceros portis* 1920, and an overview on *Praemegaceros* species in Italy. Rendiconti Lincei.

[62] Acevedo P, Cassinello J (2009). Biology, ecology and status of Iberian ibex Capra pyrenaica: a critical review and research prospectus. Mammal Review.

[63] Adhikari P (2016). Seasonal and altitudinal variation in roe deer (Capreolus pygargus tianschanicus) diet on Jeju Island, South Korea. Journal of Asia-Pacific Biodiversity.

[64] Agenbroad LD (2010). Mammuthus exilis from the California Channel Islands: height, mass, and geologic age. CIT.

[65] Agetsuma N, Agetsuma-Yanagihara Y, Takafumi H (2011). Food habits of Japanese deer in an evergreen forest: Litter-feeding deer. Mammalian Biology.

[66] Ahmad S (2020). Using an ensemble modelling approach to predict the potential distribution of Himalayan gray goral (Naemorhedus goral bedfordi) in Pakistan. Global Ecology and Conservation.

[67] Ahrestani, F. S., Heitkönig, I. M. & Prins, H. H. Diet and habitat-niche relationships within an assemblage of large herbivores in a seasonal tropical forest. *J. Trop. Ecol*., 385–394 (2012).

[68] Ahrestani, F. S., Heitkönig, I. M., Matsubayashi, H. & Prins, H. H. in *The Ecology of Large Herbivores in South and Southeast Asia* 99–120 (Springer, 2016).

[69] Aiba, K., Miura, S. & Kubo, M. O. Dental Microwear Texture Analysis in Two Ruminants, Japanese Serow (Capricornis crispus) and Sika Deer (Cervus nippon), from Central Japan. *Mammal Study* **44**, 183-192, 110 (2019).

[70] Akbari H, Habibipoor A, Mousavi J (2013). Investigation on Habitat Preferences and Group Sizes of Chinkara (Gazella bennettii) in Dareh-Anjeer Wildlife Refuge, Yazd province. Iranian Journal of Applied Ecology.

[71] Akbari H, Moradi HV, Rezaie H-R, Baghestani N (2016). Winter foraging of chinkara (Gazella bennettii shikarii) in Central Iran. Mammalia.

[72] Akersten WA, Foppe TM, Jefferson GT (1988). New source of dietary data for extinct herbivores. Quaternary Research.

[73] Akram F, Ilyas O, Haleem A (2017). Food and Feeding Habits of Indian Crested Porcupine in Pench Tiger Reserve, Madhya Pradesh, India. Ambient Sci.

[74] Al Harthi LS, Robinson MD, Mahgoub O (2008). Diets and resource sharing among livestock on the Saiq Plateau, Jebel Akhdar Mountains, Oman. International journal of ecology and environmental sciences.

[75] Alberdi MT, Prado JL, Ortiz-Jaureguizar E (1995). Patterns of body size changes in fossil and living Equini (Perissodactyla). Biological Journal of the Linnean Society.

[76] Alcover JA (2000). Vertebrate evolution and extinction on western and central Mediterranean Islands. Tropics.

[77] Alcover JA, Perez-Obiol R, Yll E-I, Bover P (1999). The diet of Myotragus balearicus Bate 1909 (Artiodactyla: Caprinae), an extinct bovid from the Balearic Islands: evidence from coprolites. Biological Journal of the Linnean Society.

[78] Ali A (2017). An assessment of food habits and altitudinal distribution of the Asiatic black bear (Ursus thibetanus) in the Western Himalayas, Pakistan. Journal of Natural History.

[79] Cornell Lab of Ornithology. All About Birds. Allaboutbirds.org (Cornell Lab of Ornithology, 2020).

[80] Myers, P. *et al*. (University of Michigan, 2019).

[81] Dantas MAT (2017). Isotopic paleoecology of the Pleistocene megamammals from the Brazilian Intertropical Region: Feeding ecology (δ13C), niche breadth and overlap. Quaternary Science Reviews.

[82] Arbouche Y, Arbouche H, Arbouche F, Arbouche R (2012). Valeur fourragere des especes prelevees par Gazella cuvieri Ogilby, 1841 au niveau du Djebel Metlili (Algerie). Archivos de Zootecnia.

[83] Arman SD, Prideaux GJ (2015). Dietary classification of extant kangaroos and their relatives (Marsupialia: Macropodoidea). Austral Ecol..

[84] Aryal A (2009). Habitat ecology of Himalayan serow (Capricornis sumatraensis ssp. thar) in Annapurna Conservation Area of Nepal. Tiger paper.

[85] Aryal A, Coogan SC, Ji W, Rothman JM, Raubenheimer D (2015). Foods, macronutrients and fibre in the diet of blue sheep (Psuedois nayaur) in the Annapurna Conservation Area of Nepal. Ecol. Evol..

[86] Asevedo L, Winck GR, Mothé D, Avilla LS (2012). Ancient diet of the Pleistocene gomphothere Notiomastodon platensis (Mammalia, Proboscidea, Gomphotheriidae) from lowland mid-latitudes of South America: Stereomicrowear and tooth calculus analyses combined. Quaternary International.

[87] Asensio, B. A., Méndez, J. R. & Prado, J. L. Patterns of body-size change in large mammals during the Late Cenozoic in the Northwestern Mediterranean. 464-479 (Museo Arqueológico Regional) (2004).

[88] Ashraf N, Anwar M, Hussain I, Nawaz MA (2014). Competition for food between the markhor and domestic goat in Chitral, Pakistan. Turkish Journal of Zoology.

[89] Ashraf N (2017). Seasonal variation in the diet of the grey goral (Naemorhedus goral) in Machiara National Park (MNP), Azad Jammu and Kashmir, Pakistan. Mammalia.

[90] The Australian Museum. Animal Fact Sheets. www.australian.museum/learn (New South Wales Government, New South Wales, 2019).

[91] Avaliani, N., Chunashvili, T., Sulamanidze, G. & Gurchiani, I. Supporting conservation of West Caucasian Tur (Capra caucasica) in Georgia. Conservation Leadership Pgoramme. *Project No: 400206* (2007).

[92] Baamrane MAA (2012). Assessment of the food habits of the Moroccan dorcas gazelle in M’Sabih Talaa, west central Morocco, using the trn L approach. PLoS One.

[93] Bailey M, Petrie SA, Badzinski SS (2008). Diet of mute swans in lower Great Lakes coastal marshes. The Journal of wildlife Management.

[94] Ballari SA, Barrios‐García MN (2014). A review of wild boar Sus scrofa diet and factors affecting food selection in native and introduced ranges. Mammal Review.

[95] Barboza P, Hume I (1992). Digestive tract morphology and digestion in the wombats (Marsupialia: Vombatidae). Journal of Comparative Physiology B.

[96] Bargo MS (2001). The ground sloth Megatherium americanum: skull shape, bite forces, and diet. Acta Palaeontologica Polonica.

[97] Bargo MS, Vizcaíno SF (2008). Paleobiology of Pleistocene ground sloths (Xenarthra, Tardigrada): biomechanics, morphogeometry and ecomorphology applied to the masticatory apparatus. Ameghiniana.

[98] Bargo MS, Toledo N, Vizcaíno SF (2006). Muzzle of South American Pleistocene ground sloths (Xenarthra, Tardigrada). J. Morphol..

[99] Barreto, G. R. & Quintana, R. D. *in Capybara*. (Springer, 2013).

[100] Baskaran N, Kannan V, Thiyagesan K, Desai AA (2011). Behavioural ecology of four-horned antelope (Tetracerus quadricornis de Blainville, 1816) in the tropical forests of southern India. Mammalian Biology.

[101] Baskaran N, Ramkumaran K, Karthikeyan G (2016). Spatial and dietary overlap between blackbuck (Antilope cervicapra) and feral horse (Equus caballus) at Point Calimere Wildlife Sanctuary, Southern India: Competition between native versus introduced species. Mammalian Biology.

[102] Basumatary SK, Singh H, McDonald HG, Tripathi S, Pokharia AK (2019). Modern botanical analogue of endangered Yak (Bos mutus) dung from India: Plausible linkage with extant and extinct megaherbivores. PLoS One.

[103] Bedaso ZK, Wynn JG, Alemseged Z, Geraads D (2013). Dietary and paleoenvironmental reconstruction using stable isotopes of herbivore tooth enamel from middle Pliocene Dikika, Ethiopia: Implication for Australopithecus afarensis habitat and food resources. J. Hum. Evol..

[104] Benamor N, Bounaceur F, Baha M, Aulagnier S (2019). First data on the seasonal diet of the vulnerable Gazella cuvieri (Mammalia: Bovidae) in the Djebel Messaâd forest, northern Algeria. Folia Zoologica.

[105] Bennett, C. V. & Goswami, A. Statistical support for the hypothesis of developmental constraint in marsupial skull evolution. *BMC Biol*. **11** (2013). 10.1186/1741-7007-11-52 PMC3660189 23622087

[106] Bergmann GT, Craine JM, Robeson MS, Fierer N (2015). Seasonal shifts in diet and gut microbiota of the American bison (Bison bison). PLoS One.

[107] Bhat SA, Telang S, Wani MA, Sheikh KA (2015). Food habits of Nilgai (Boselaphus tragocamelus) in Van Vihar National Park, Bhopal, Madhya Pradesh, India. Biomedical and Pharmacology Journal.

[108] Bhattacharya, T., Kittur, S., Sathyakumar, S. & Rawat, G. Diet overlap between wild ungulates and domestic livestock in the greater Himalaya: implications for management of grazing practices in *Proceedings of the Zoological Society*. 11-21 (Springer).

[109] Bibi F, Kiessling W (2015). Continuous evolutionary change in Plio-Pleistocene mammals of eastern. Africa. Proc. Natl. Acad. Sci. USA.

[110] Biknevicius, A. R., McFarlane, D. A. & MacPhee, R. D. E. Body size in Amblyrhiza inundata (Rodentia, Caviomorpha), an extinct megafaunal rodent from the Anguilla Bank, West Indies: estimates and implications. American Museum novitates; no. 3079 (1993).

[111] Cornell Lab of Ornithology. Birds of the World. https://birdsoftheworld.org/bow Cornell Lab of Ornithology (2020).

[112] Biswas J (2011). The enigmatic Arunachal macaque: its biogeography, biology and taxonomy in Northeastern India. Am. J. Primatol..

[113] Bocherens H (2017). Isotopic insight on paleodiet of extinct Pleistocene megafaunal Xenarthrans from Argentina. Gondwana Research.

[114] Boeskorov GG (2011). Woolly rhino discovery in the lower Kolyma River. Quaternary Science Reviews.

[115] Bojarska K, Selva N (2012). Spatial patterns in brown bear Ursus arctos diet: the role of geographical and environmental factors. Mammal Review.

[116] Bon R, Rideau C, Villaret J-C, Joachim J (2001). Segregation is not only a matter of sex in Alpine ibex, Capra ibex ibex. Anim. Behav..

[117] Bond WJ, Silander JA, Ranaivonasy J, Ratsirarson J (2008). The antiquity of Madagascar’s grasslands and the rise of C4 grassy biomes. Journal of Biogeography.

[118] Borgnia M, Vilá BL, Cassini MH (2010). Foraging ecology of Vicuña, Vicugna vicugna, in dry Puna of Argentina. Small Rumin. Res..

[119] Bowman DM, Murphy BP, McMahon CR (2010). Using carbon isotope analysis of the diet of two introduced Australian megaherbivores to understand Pleistocene megafaunal extinctions. Journal of Biogeography.

[120] Bradford MG, Dennis AJ, Westcott DA (2008). Diet and dietary preferences of the southern cassowary (Casuarius casuarius) in North Queensland, Australia. Biotropica.

[121] Bradham JL, DeSantis LR, Jorge MLS, Keuroghlian A (2018). Dietary variability of extinct tayassuids and modern white-lipped peccaries (Tayassu pecari) as inferred from dental microwear and stable isotope analysis. Palaeogeography, Palaeoclimatology, Palaeoecology.

[122] Bravo-Cuevas VM, Rivals F, Priego-Vargas J (2017). Paleoecology (δ13C and δ18O stable isotopes analysis) of a mammalian assemblage from the late Pleistocene of Hidalgo, central Mexico and implications for a better understanding of environmental conditions in temperate North America (18°–36° N Lat.). Palaeogeography, Palaeoclimatology, Palaeoecology.

[123] Bravo-Cuevas VM, Jiménez-Hidalgo E, Perdoma MAC, Priego-Vargas J (2013). Taxonomy and notes on the paleobiology of the late Pleistocene (Rancholabrean) antilocaprids (Mammalia, Artiodactyla, Antilocapridae) from the state of Hidalgo, central Mexico. Revista mexicana de Ciencias Geológicas.

[124] Buchsbaum R, Wilson J, Valiela I (1986). Digestibility of plant constitutents by Canada Geese and Atlantic Brant. Ecology.

[125] Buckland, R. & Guy, G. *Goose Production Systems*, http://www.fao.org/3/y4359e/y4359e00.htm#Contents (2002).

[126] Burness GP, Diamond J, Flannery T (2001). Dinosaurs, dragons, and dwarfs: the evolution of maximal body size. Proc. Natl. Acad. Sci. USA.

[127] Burton J, Hedges S, Mustari A (2005). The taxonomic status, distribution and conservation of the lowland anoa Bubalus depressicornis and mountain anoa Bubalus quarlesi. Mammal Review.

[128] Butler K, Louys J, Travouillon K (2014). Extending dental mesowear analyses to Australian marsupials, with applications to six Plio-Pleistocene kangaroos from southeast Queensland. Palaeogeography, Palaeoclimatology, Palaeoecology.

[129] Cain JW, Avery MM, Caldwell CA, Abbott LB, Holechek JL (2017). Diet composition, quality and overlap of sympatric American pronghorn and gemsbok. Wildlife Biology.

[130] Campbell JL, Eisemann JH, Williams CV, Glenn KM (2000). Description of the Gastrointestinal Tract of Five Lemur Species: Propithecus tattersalli, Propithecus verreauxicoquereli, Varecia variegata, Hapalemur griseus, and Lemur catta. Am. J. Primatol..

[131] Carey SP (2011). A diverse Pleistocene marsupial trackway assemblage from the Victorian Volcanic Plains, Australia. Quaternary Science Reviews.

[132] Cartelle C, Hartwig WC (1996). A new extinct primate among the Pleistocene megafauna of Bahia, Brazil. Proc. Natl. Acad. Sci. USA.

[133] Cassini, G. H., Cerdeño, E., Villafañe, A. L. & Muñoz, N. A. Paleobiology of Santacrucian native ungulates (Meridiungulata: Astrapotheria, Litopterna and Notoungulata) in *Early Miocene Paleobiology in Patagonia/Vizcaíno* (Cambridge University Press) (2012).

[134] Cerdeño E (1998). Diversity and evolutionary trends of the Family Rhinocerotidae (Perissodactyla). Palaeogeography, Palaeoclimatology, Palaeoecology.

[135] Cerling TE, Viehl K (2004). Seasonal diet changes of the forest hog (*Hylochoerus meinertzhageni* Thomas) based on the carbon isotopic composition of hair. African Journal of Ecology.

[136] Chaiyarat R, Saengpong S, Tunwattana W, Dunriddach P (2017). Habitat and food utilization by banteng (Bos javanicus d’Alton, 1823) accidentally introduced into the Khao Khieo-Khao Chomphu Wildlife Sanctuary, Thailand. Mammalia.

[137] Chen Y (2019). Activity Rhythms of Coexisting Red Serow and Chinese Serow at Mt. Gaoligong as Identified by Camera Traps. Animals.

[138] Choudhury A (1994). The decline of the wild water buffalo in north-east India. Oryx.

[139] Christiansen P (1999). What size were *Arctodus simus* and *Ursus spelaeus* (Carnivora: Ursidae)?. Annales Zoologici Fennici.

[140] Christiansen P (2004). Body size in proboscideans, with notes on elephant metabolism. Zoological journal of the Linnean Society.

[141] Chritz KL (2009). Palaeobiology of an extinct Ice Age mammal: Stable isotope and cementum analysis of giant deer teeth. Palaeogeography, Palaeoclimatology, Palaeoecology.

[142] Clarke SJ, Miller GH, Fogel ML, Chivas AR, Murray-Wallace CV (2006). The amino acid and stable isotope biogeochemistry of elephant bird (Aepyornis) eggshells from southern Madagascar. Quaternary Science Reviews.

[143] Clauss M (2004). The potential interplay of posture, digestive anatomy, density of ingesta and gravity in mammalian herbivores: Why sloths do not rest upside down. Mammal Review.

[144] Clauss M (2003). The maximum attainable body size of herbivorous mammals: morphophysiological constraints on foregut, and adaptations of hindgut fermenters. Oecologia.

[145] Clauss M, Hummel J, Vercammen F, Streich WJ (2005). Observations on the Macroscopic Digestive Anatomy of the Himalayan Tahr (Hemitragus jemlahicus). Anatomia Histologia Embryologia.

[146] Clench, M. H. & Mathias, J. R. The avian cecum: a review. *The Wilson Bulletin*, 93–121 (1995).

[147] Cobb MA, KHelling H, Pyle B (2012). Summer diet and feeding location selection patterns of an irrupting mountain goat population on Kodiak Island, Alaska. Biennial Symposium of the Northern Wild Sheep and Goat Council.

[148] Codron D, Brink JS, Rossouw L, Clauss M (2008). The evolution of ecological specialization in southern African ungulates: competition- or physical environmental turnover?. Oikos.

[149] Codron D, Clauss M, Codron J, Tütken T (2018). Within trophic level shifts in collagen–carbonate stable carbon isotope spacing are propagated by diet and digestive physiology in large mammal herbivores. Ecol. Evol..

[150] Comparatore V, Yagueddú C (2007). Diet of the Greater Rhea (Rhea americana) in an agroecosystem of the Flooding Pampa, Argentina. Ornitologia Neotropical.

[151] Cooke SB (2011). Paleodiet of extinct platyrrhines with emphasis on the Caribbean forms: three-dimensional geometric morphometrics of mandibular second molars. The Anatatomical Record.

[152] Coombs MC (1983). Large mammalian clawed herbivores: a comparative study. Transactions of the American Philosophical Society.

[153] Cope ED (1883). The extinct rodentia of North America. The American Naturalist.

[154] Corona A, Ubilla Gutierrez M, Perea Negreira D (2019). New records and diet reconstruction using dental microwear analysis for Neolicaphrium recens Frenguelli, 1921 (Litopterna, Proterotheriidae). Andean Geology, 2019.

[155] Craine JM, Towne EG, Miller M, Fierer N (2015). Climatic warming and the future of bison as grazers. Sci. Rep..

[156] Cransac, N., Valet, G., Cugnasse, J.-M. & Rech, J. Seasonal diet of mouflon (*Ovis gmelini*): comparison of population sub-units and sex-age classes. *Revue d'écologie* (1997).

[157] Creese S, Davies SJ, Bowen BJ (2019). Comparative dietary analysis of the black-flanked rock-wallaby (Petrogale lateralis lateralis), the euro (Macropus robustus erubescens) and the feral goat (Capra hircus) from Cape Range National Park, Western Australia. Aust. Mammal..

[158] Croitor R (2016). Systematical position and paleoecology of the endemic deer *Megaceroides algericus* Lydekker, 1890 (Cervidae, Mammalia) from the late Pleistocene-early Holocene of North Africa. Geobios.

[159] Croitor R, Bonifay M-F, Brugal J-P (2008). Systematic revision of the endemic deer Haploidoceros n. gen. mediterraneus (Bonifay, 1967)(Mammalia, Cervidae) from the Middle Pleistocene of Southern France. Paläontologische Zeitschrift.

[160] Cromsigt JPGM, Kemp YJM, Rodrigues E, Kivit H (2017). Rewilding Europe’s large grazer community: how functionally diverse are the diets of European bison, cattle, and horses?. Restoration Ecology.

[161] Crowley, B. E. & Godfrey, L. R. in *Leaping Ahead* 173-182 (Springer, 2012).

[162] Crowley BE, Samonds KE (2013). Stable carbon isotope values confirm a recent increase in grasslands in northwestern Madagascar. The Holocene.

[163] Crowley BE, Godfrey LR, Irwin MT (2011). A glance to the past: subfossils, stable isotopes, seed dispersal, and lemur species loss in southern Madagascar. Am. J. Primatol..

[164] Cunningham PL, Wacher T (2009). Changes in the distribution, abundance and status of Arabian Sand Gazelle (Gazella subgutturosa marica) in Saudi Arabia: a review. Mammalia.

[165] Czerwonogora A, Fariña RA, Tonni EP (2011). Diet and isotopes of Late Pleistocene ground sloths: first results for Lestodon and Glossotherium (Xenarthra, Tardigrada). Neues Jahrbuch fur Geologie und Paleontologie - Abhandlungen.

[166] Domanov TA (2013). Musk deer Moschus moschiferus nutrition in the Tukuringra Mountain Range, Russian Far East, during the snow season. Russian Journal of Theriology.

[167] Dantas, M. A. T. & Cozzuol, M. A. in *Marine Isotope Stage 3 in Southern South America, 60 KA B.P.-30 KA B.P*. (eds Germán Mariano Gasparini, Jorge Rabassa, Cecilia Deschamps, & Eduardo Pedro Tonni) 207-226 (Springer International Publishing, 2016).

[168] Dantas MAT (2013). Paleoecology and radiocarbon dating of the Pleistocene megafauna of the Brazilian Intertropical Region. Quaternary Research.

[169] Dantas, M. A. T. *et al*. Isotopic paleoecology (δ 13C) of mesoherbivores from Late Pleistocene of Gruta da Marota, Andaraí, Bahia, Brazil. *Hist. Biol*., 1–9 (2019).

[170] Dantas MAT (2020). Isotopic paleoecology (δ13C) from mammals from IUIU/BA and paleoenvironmental reconstruction (δ13C, δ18O) for the Brazilian intertropical region through the late Pleistocene. Quaternary Science Reviews.

[171] Davids, A. H. *Estimation of genetic distances and heterosis in three ostrich (Struthio camelus) breeds for the improvement of productivity*, Stellenbosch: University of Stellenbosch, (2011).

[172] Davies, P. & Lister, A. M. in *The World of Elephants International Congress* 479-480 (International Congress, Rome 2001, 2001).

[173] Dawson L (2006). An ecophysiological approach to the extinction of large marsupial herbivores in middle and late Pleistocene Australia. Alcheringa: An Australasian Journal of Palaeontology.

[174] Dawson, T. J. *et al*. in *Fauna of Australia* (eds D. W. Walton & B. J. Richardson) (AGPS Canberra, 1989).

[175] De Iuliis G, Bargo MS, Vizcaíno SF (2000). Variation in skull morphology and mastication in the fossil giant armadillos Pampatherium spp. and allied genera (Mammalia: Xenarthra: Pampatheriidae), with comments on their systematics and distribution. Journal of Vertebrate Paleontology.

[176] de Oliveira AM, Santos CMD (2018). Functional morphology and paleoecology of Pilosa (Xenarthra, Mammalia) based on a two‐dimensional geometric Morphometrics study of the Humerus. J. Morphol..

[177] de Oliveira K (2020). Fantastic beasts and what they ate: Revealing feeding habits and ecological niche of late Quaternary Macraucheniidae from South America. Quaternary Science Reviews.

[178] DeSantis LRG, Field JH, Wroe S, Dodson JR (2017). Dietary responses of Sahul (Pleistocene Australia–New Guinea) megafauna to climate and environmental change. Paleobiology.

[179] Desbiez ALJ, Santos SA, Alvarez JM, Tomas WM (2011). Forage use in domestic cattle (Bos indicus), capybara (Hydrochoerus hydrochaeris) and pampas deer (Ozotoceros bezoarticus) in a seasonal Neotropical wetland. Mammalian Biology.

[180] Dierenfeld E, Hintz H, Robertson J, Van Soest P, Oftedal O (1982). Utilization of bamboo by the giant panda. The Journal of Nutrition.

[181] Djagoun C, Codron D, Sealy J, Mensah G, Sinsin B (2013). Stable carbon isotope analysis of the diets of West African bovids in Pendjari Biosphere Reserve, Northern Benin. African Journal of Wildlife Research.

[182] Domingo L, Prado JL, Alberdi MT (2012). The effect of paleoecology and paleobiogeography on stable isotopes of Quaternary mammals from South America. Quaternary Science Reviews.

[183] Dong W (2014). Late Pleistocene mammalian fauna from Wulanmulan Paleolithic Site, Nei Mongol, China. Quaternary International.

[184] Doody JS, Sims RA, Letnic M (2007). Environmental Manipulation to Avoid a Unique Predator: Drinking Hole Excavation in the Agile Wallaby, Macropus agilis. Ethology.

[185] Dookia, S. & Jakher, G. R. Food and Feeding Habit of Indian Gazelle (Gazella bennettii), in the Thar Desert of Rajasthan. *The Indian Forester* **133** (2007).

[186] Downer CC (2001). Observations on the diet and habitat of the mountain tapir (Tapirus pinchaque). J. Zool..

[187] Dunning, J. B. Jr *CRC handbook of avian body masses*. (CRC press, 2007).

[188] Dunstan H, Florentine SK, Calviño-Cancela M, Westbrooke ME, Palmer GC (2013). Dietary characteristics of Emus (Dromaius novaehollandiae) in semi-arid New South Wales, Australia, and dispersal and germination of ingested seeds. Emu-Austral Ornithology.

[189] Endo, Y., Takada, H. & Takatsuki, S. Comparison of the Food Habits of the Sika Deer (*Cervus nippon*), the Japanese Serow (*Capricornis crispus*), and the Wild Boar (*Sus scrofa*), Sympatric Herbivorous Mammals from Mt. Asama, Central Japan. *Mammal Study* **42**, 131-140, 110 (2017).

[190] Espunyes J (2019). Seasonal diet composition of Pyrenean chamois is mainly shaped by primary production waves. PLoS One.

[191] Evans MC, Macgregor C, Jarman PJ (2006). Diet and feeding selectivity of common wombats. Wildlife Research.

[192] Faith JT (2011). Late Quaternary dietary shifts of the Cape grysbok (Raphicerus melanotis) in southern Africa. Quaternary Research.

[193] Faith JT (2014). Late Pleistocene and Holocene mammal extinctions on continental Africa. Earth-Science Reviews.

[194] Faith JT, Behrensmeyer AK (2013). Climate change and faunal turnover: testing the mechanics of the turnover-pulse hypothesis with South African fossil data. Paleobiology.

[195] Faith JT, Thompson JC (2013). Fossil evidence for seasonal calving and migration of extinct blue antelope (Hippotragus leucophaeus) in southern Africa. Journal of Biogeography.

[196] Faith JT (2012). New perspectives on middle Pleistocene change in the large mammal faunas of East Africa: Damaliscus hypsodon sp. nov. (Mammalia, Artiodactyla) from Lainyamok, Kenya. Palaeogeography, Palaeoclimatology, Palaeoecology.

[197] Fanelli F, Palombo MR, Pillola GL, Ibba A (2007). Tracks and trackways of “Praemegaceros” cazioti (Depéret, 1897) (Artiodactyla, Cervidae) in Pleistocene coastal deposits from Sardinia (Western Mediterranean, Italy). Bollettino della Società Paleontologica Italiana.

[198] Farhadinia MS (2009). Goitered Gazelle, Gazella subgutturosa: its habitat preference and conservation needs in Miandasht Wildlife Refuge, north-eastern Iran (Mammalia: Artiodactyla). Zoology in the middle east.

[199] Fariña RA, Vizcaíno SF, Bargo MS (1998). Body mass estimations in Lujanian (late Pleistocene-early Holocene of South America) mammal megafauna. Mastozoología Neotropical.

[200] Feranec RS (2003). Stable isotopes, hypsodonty, and the paleodiet of Hemiauchenia (Mammalia: Camelidae): a morphological specialization creating ecological generalization. Paleobiology.

[201] Feranec R, García N, Díez J, Arsuaga J (2010). Understanding the ecology of mammalian carnivorans and herbivores from Valdegoba cave (Burgos, northern Spain) through stable isotope analysis. Palaeogeography, Palaeoclimatology, Palaeoecology.

[202] Fernández-Olalla M, Martínez-Jauregui M, Perea R, Velamazán M, San Miguel A (2016). Threat or opportunity? Browsing preferences and potential impact of Ammotragus lervia on woody plants of a Mediterranean protected area. J. Arid Environ..

[203] Ferretti MP (2008). The dwarf elephant *Palaeoloxodon mnaidriensis* from Puntali Cave, Carini (Sicily; late Middle Pleistocene): Anatomy, systematics and phylogenetic relationships. Quaternary International.

[204] Figueirido B, Soibelzon LH (2010). Inferring palaeoecology in extinct tremarctine bears (Carnivora, Ursidae) using geometric morphometrics. Lethaia.

[205] Flannery TF (1990). Pleistocene faunal loss: implications of the aftershock for Australia’s past and future. Archaeology in Oceania.

[206] Flannery TF (1993). Taxonomy of Dendrolagus goodfellowi (Macropodidae: Marsupialia) with description of a new subspecies. Records of the Australian Museum.

[207] Flannery TF (1999). The Pleistocene mammal fauna of Kelangurr Cave, central montane Irian Jaya, Indonesia. Records of the Western Australian Museum.

[208] Flannery, T. F., Martin, R. & Szalay, A. *Tree kangaroos: a curious natural history*. (Reed Books, 1996).

[209] Fleagle, J. G. & Gilbert, C. C. *Elwyn Simons: a search for origins*. (Springer Science & Business Media, 2007).

[210] Foerster CR, Vaughan C (2015). Diet and foraging behavior of a female Baird’s tapir (Tapirus bairdi) in a Costa Rican lowland rainforest. Cuadernos de Investigación UNED.

[211] Fooden J (2007). Systematic review of the Barbary Macaque, *Macaca sylvanus* (Linnaeus, 1758). Fieldiana Zoology.

[212] Forasiepi AM (2016). Exceptional skull of Huayqueriana (Mammalia, Litopterna, Macraucheniidae) from the late Miocene of Argentina: anatomy, systematics, and paleobiological implications. Bulletin of the American Museum of Natural History.

[213] França LdM (2014). Chronology and ancient feeding ecology of two upper Pleistocene megamammals from the Brazilian Intertropical Region. Quaternary Science Reviews.

[214] França LdM (2015). Review of feeding ecology data of Late Pleistocene mammalian herbivores from South America and discussions on niche differentiation. Earth-Science Reviews.

[215] France CA, Zelanko PM, Kaufman AJ, Holtz TR (2007). Carbon and nitrogen isotopic analysis of Pleistocene mammals from the Saltville Quarry (Virginia, USA): Implications for trophic relationships. Palaeogeography, Palaeoclimatology, Palaeoecology.

[216] Fuller BT (2020). Pleistocene paleoecology and feeding behavior of terrestrial vertebrates recorded in a pre-LGM asphaltic deposit at Rancho La Brea, California. Palaeogeography, Palaeoclimatology, Palaeoecology.

[217] Furley, C. W. Potential Use of Gazelles for Game Ranching in the Arabian Peninsula (This lecture was delivered at the Agro-Gulf Exhibition and Conference, Abu Dhabi, 1983.).

[218] Gad SD, Shyama SK (2011). Diet composition and quality in Indian bison (Bos gaurus) based on fecal analysis. Zoolog. Sci..

[219] Gagnon M, Chew AE (2000). Dietary preferences in extant African Bovidae. J. Mammal..

[220] García A, Carretero EM, Dacar MA (2008). Presence of Hippidion at two sites of western Argentina: Diet composition and contribution to the study of the extinction of Pleistocene megafauna. Quaternary International.

[221] García‐Rangel S (2012). Andean bear Tremarctos ornatus natural history and conservation. Mammal Review.

[222] Gardner PC, Ridge S, Wern JGE, Goossens B (2019). The influence of logging upon the foraging behaviour and diet of the endangered Bornean banteng. Mammalia.

[223] Garitano-Zavala A, Nadal J, Ávila P (2003). The feeding ecology and digestive tract morphometry of two sympatric tinamous of the high plateau of the Bolivian Andes: the Ornate Tinamou (Nothoprocta ornata) and the Darwin’s Nothura (Nothura darwinii). Ornitología Neotropical.

[224] Garrett ND (2015). Stable isotope paleoecology of Late Pleistocene Middle Stone Age humans from the Lake Victoria basin, Kenya. J. Hum. Evol..

[225] Gasparini GM, Kerber L, Oliveira EV (2009). Catagonus stenocephalus (Lund in Reinhardt, 1880)(Mammalia, Tayassuidae) in the Touro Passo Formation (Late Pleistocene), Rio Grande do Sul, Brazil. Taxonomic and palaeoenvironmental comments. Neues Jahrbuch für Geologie und Paläontologie-Abhandlungen.

[226] Gasparini GM, Soibelzon E, Zurita AE, Miño-Boilini AR (2010). A review of the Quaternary Tayassuidae (Mammalia, Artiodactyla) from the Tarija Valley, Bolivia. Alcheringa: An Australasian Journal of Palaeontology.

[227] Gautier-Hion, A. & Gautier, J.-P. Cephalophus ogilbyi crusalbum Grubb 1978, described from coastal Gabon, is quite common in the Forêt des Abeilles, Central Gabon. *Revue d’Écologie* **2** (1994).

[228] Gautier-Hion A, Emmons LH, Dubost G (1980). A comparison of the diets of three major groups of primary consumers of Gabon (primates, squirrels and ruminants). Oecologia.

[229] Gavashelishvili A (2004). Habitat selection by East Caucasian tur (Capra cylindricornis). Biol. Conserv..

[230] Gebremedhin B (2016). DNA Metabarcoding Reveals Diet Overlap between the Endangered Walia Ibex and Domestic Goats - Implications for Conservation. PLoS One.

[231] Geist, V. *Deer of the world: their evolution, behaviour, and ecology*. (Stackpole books, 1998).

[232] Ghosh, A., Thakur, M., Singh, S. K., Sharma, L. K. & Chandra, K. Gut microbiota suggests dependency of Arunachal Macaque (Macaca munzala) on anthropogenic food in Western Arunachal Pradesh, Northeastern India: Preliminary findings. *Global Ecology and Conservation*, e01030 (2020).

[233] Giles FH (1937). The riddle of Cervus schomburgki. Journal of the Siam Society Natural History Supplement.

[234] Gill, F. B. *Ornithology*. (W.H. Freeman and Company, 2001).

[235] Gillette, D. D. & Ray, C. E. *Glyptodonts of North America*. Vol. 40 (1981).

[236] Gingerich, P. D. Land-to-sea transition in early whales: evolution of Eocene Archaeoceti (Cetacea) in relation to skeletal proportions and locomotion of living semiaquatic mammals. *Paleobiology* **29**, 429–454, 10.1666/0094-8373(2003)029<0429:LTIEWE>2.0.CO;2 (2003).

[237] Giri S, Aryal A, Koirala R, Adhikari B, Raubenheimer D (2011). Feeding ecology and distribution of Himalayan serow (Capricornis thar) in Annapurna Conservation Area, Nepal. World Journal of Zoology.

[238] Godfrey LR (2004). Dental use wear in extinct lemurs: evidence of diet and niche differentiation. J. Hum. Evol..

[239] González-Guarda E (2017). Late Pleistocene ecological, environmental and climatic reconstruction based on megafauna stable isotopes from northwestern Chilean Patagonia. Quaternary Science Reviews.

[240] Gazzolo, C. & Barrio, J. Feeding ecology of taruca (Hippocamelus antisensis) populations during the rainy and dry seasons in Central Peru. *International Journal of Zoology* **2016** (2016).

[241] Grass, A. D. *Inferring lifestyle and locomotor habits of extinct sloths through scapula morphology and implications for convergent evolution in extant sloths* PhD thesis, Graduate College of the University of Iowa, (2014).

[242] Gray GG, Simpson CD (1980). Ammotragus lervia. Mammalian Species.

[243] Green JL (2009). Dental microwear in the orthodentine of the Xenarthra (Mammalia) and its use in reconstructing the palaeodiet of extinct taxa: the case study of Nothrotheriops shastensis (Xenarthra, Tardigrada, Nothrotheriidae). Zoological Journal of the Linnean Society.

[244] Green JL, Kalthoff DC (2015). Xenarthran dental microstructure and dental microwear analyses, with new data for Megatherium americanum (Megatheriidae). J. Mammal..

[245] Green K, Davis N, Robinson W (2015). The diet of the common wombat (Vombatus ursinus) above the winter snowline in the decade following a wildfire. Aust. Mammal..

[246] Green JL, DeSantis LRG, Smith GJ (2017). Regional variation in the browsing diet of Pleistocene Mammut americanum (Mammalia, Proboscidea) as recorded by dental microwear textures. Palaeogeography, Palaeoclimatology, Palaeoecology.

[247] Grignolio S, Parrini F, Bassano B, Luccarini S, Apollonio M (2003). Habitat selection in adult males of Alpine ibex. Capra ibex ibex. Folia Zoologica-Praha.

[248] Gröcke DR (1997). Distribution of C3 and C4 plants in the late Pleistocene of South Australia recorded by isotope biogeochemistry of collagen in megafauna. Australian Journal of Botany.

[249] Gröcke D, Bocherens H (1996). Isotopic investigation of an Australian island environment. *Comptes Rendus de l’Academie des Sciences. Serie 2*. Sciences de la Terre et des Planetes.

[250] Groves CP, Leslie DM (2011). Rhinoceros sondaicus (Perissodactyla: Rhinocerotidae). Mammalian Species.

[251] Guerrero-Cardenas, I., Gallina, S., del Rio, P. C. M., Cardenas, S. A. & Orduña, R. R. Composición y selección de la dieta del borrego cimarrón (Ovis canadensis) en la Sierra El Mechudo, Baja California Sur, México. *Therya* (2016).

[252] Hadjisterkotis E, Reese DS (2008). Considerations on the potential use of cliffs and caves by the extinct endemic late pleistocene hippopotami and elephants of Cyprus. European Journal of Wildlife Research.

[253] Haleem A, Ilyas O (2018). Food and Feeding Habits of Gaur (*Bos gaurus*) in Highlands of Central India: A Case Study at Pench Tiger Reserve, Madhya Pradesh (India). Zoolog. Sci..

[254] Halenar LB (2011). Reconstructing the Locomotor Repertoire of Protopithecus brasiliensis. II. Forelimb Morphology. The Anatomical Record.

[255] Halenar, L. B. *Paleobiology of Protopithecus brasiliensis, a plus-size Pleistocene platyrrhine from Brazil*, City University of New York, (2012).

[256] Hamilton WJ, Buskirk R, Buskirk WH (1977). Intersexual dominance and differential mortality of Gemsbok *Oryx gazella* at Namib Desert waterholes. Madoqua.

[257] Hansen RM (1978). Shasta ground sloth food habits, Rampart Cave, Arizona. Paleobiology.

[258] Hansford JP, Turvey ST (2018). Unexpected diversity within the extinct elephant birds (Aves: Aepyornithidae) and a new identity for the world’s largest bird. Royal Society open science.

[259] Harris JM, Cerling TE (2002). Dietary adaptations of extant and Neogene African suids. J. Zool..

[260] Hartwig WC, Cartelle C (1996). A complete skeleton of the giant South American primate Protopithecus. Nature.

[261] Heinen JH, van Loon EE, Hansen DM, Kissling WD (2018). Extinction‐driven changes in frugivore communities on oceanic islands. Ecography.

[262] Hempson GP, Archibald S, Bond WJ (2015). A continent-wide assessment of the form and intensity of large mammal herbivory in Africa. Science.

[263] Henry O, Feer F, Sabatier D (2000). Diet of the lowland tapir (Tapirus terrestris L.) in French Guiana. Biotropica.

[264] Herd RM, Dawson TJ (1984). Fiber digestion in the emu, Dromaius novaehollandiae, a large bird with a simple gut and high rates of passage. Physiol. Zool..

[265] Herridge VL, Lister AM (2012). Extreme insular dwarfism evolved in a mammoth. Proc. R. Soc. B..

[266] Heywood J (2010). Functional anatomy of bovid upper molar occlusal surfaces with respect to diet. J. Zool..

[267] Hofreiter M (2000). A molecular analysis of ground sloth diet through the last glaciation. Mol. Ecol..

[268] Hollis C, Robertshaw J, Harden R (1986). Ecology of the swamp wallaby (Wallabia-Bicolor) in northeastern New-South-Wales. 1. Diet. Wildlife Research.

[269] Hope G, Flannery T (1993). A preliminary report of changing Quaternary mammal faunas in subalpine New Guinea. Quaternary Research.

[270] Hou R (2018). Seasonal variation in diet and nutrition of the northern‐most population of Rhinopithecus roxellana. Am. J. Primatol..

[271] Huffman, B. *Rucervus schomburgki*. Ultimate Ungulate. http://www.ultimateungulate.com/Artiodactyla/Rucervus_schomburgki.html (2020).

[272] Hullot M, Antoine P-O, Ballatore M, Merceron G (2019). Dental microwear textures and dietary preferences of extant rhinoceroses (Perissodactyla, Mammalia). Mammal Research.

[273] Hume JP (2006). The history of the Dodo Raphus cucullatus and the penguin of Mauritius. Hist. Biol..

[274] Hummel J (2008). Fluid and particle retention in the digestive tract of the addax antelope (Addax nasomaculatus)—Adaptations of a grazing desert ruminant. Comparative Biochemistry and Physiology Part A: Molecular & Integrative Physiology.

[275] Iribarren C, Kotler BP (2012). Foraging patterns of habitat use reveal landscape of fear of Nubian ibex Capra nubiana. Wildlife Biology.

[276] Ismail K, Kamal K, Plath M, Wronski T (2011). Effects of an exceptional drought on daily activity patterns, reproductive behaviour, and reproductive success of reintroduced Arabian oryx (Oryx leucoryx). J. Arid Environ..

[277] IUCN Redlist. The International Union for the Conservation of Nature 2018.

[278] Iwaniuk AN, Pellis SM, Whishaw IQ (2000). The relative importance of body size, phylogeny, locomotion, and diet in the evolution of forelimb dexterity in fissiped carnivores (Carnivora). Can. J. Zool..

[279] Iwase A, Hashizume J, Izuho M, Takahashi K, Sato H (2012). Timing of megafaunal extinction in the late Late Pleistocene on the Japanese Archipelago. Quaternary International.

[280] Jackson J (1977). The annual diet of the fallow deer (Dama dama) in the New Forest, Hampshire, as determined by rumen content analysis. J. Zool..

[281] Janis, C. M., Napoli, J. G., Billingham, C. & Martín-Serra, A. Proximal humerus morphology indicates divergent patterns of locomotion in extinct giant kangaroos. *J. Mamm. Evol*., 1–21 (2020).

[282] Jankowski NR, Gully GA, Jacobs Z, Roberts RG, Prideaux GJ (2016). A late Quaternary vertebrate deposit in Kudjal Yolgah Cave, south‐western Australia: refining regional late Pleistocene extinctions. Journal of Quaternary Science.

[283] Janssen R (2016). Tooth enamel stable isotopes of Holocene and Pleistocene fossil fauna reveal glacial and interglacial paleoenvironments of hominins in Indonesia. Quaternary Science Reviews.

[284] Al-Jassim, R. & Hogan, J. in *Proc. 3rd ISOCARD Conference. Keynote presentations*. *29th January–1st February*. 75–86.

[285] Jhala, Y. V. & Isvaran, K. in *The Ecology of Large Herbivores in South and Southeast Asia* 151–176 (Springer, 2016).

[286] Jiménez-Hidalgo E (2019). Species diversity and paleoecology of Late Pleistocene horses from southern Mexico. Frontiers in Ecology and Evolution.

[287] Johnson, C. *Australia’s mammal extinctions: a 50,000-year history*. (Cambridge University Press, 2006).

[288] Johnson CN, Prideaux GJ (2004). Extinctions of herbivorous mammals in the late Pleistocene of Australia in relation to their feeding ecology: no evidence for environmental change as cause of extinction. Austral Ecol..

[289] Jones T (2005). The Highland Mangabey *Lophocebus kipunji*: A New Species of African Monkey. Science.

[290] Jones KE (2009). PanTHERIA: a species‐level database of life history, ecology, and geography of extant and recently extinct mammals: Ecological Archives E090‐184. Ecology.

[291] Jones DB, DeSantis LR (2016). Dietary ecology of the extinct cave bear: evidence of omnivory as inferred from dental microwear textures. Acta Palaeontologica Polonica.

[292] Jungers WL, Godfrey LR, Simons EL, Chatrath PS (1997). Phalangeal curvature and positional behavior in extinct sloth lemurs (Primates, Palaeopropithecidae). Proc. Natl. Acad. Sci. USA.

[293] Jungers WL (2005). The hands and feet of Archaeolemur: metrical affinities and their functional significance. J. Hum. Evol..

[294] Kaczensky P (2017). Stable isotopes reveal diet shift from pre-extinction to reintroduced Przewalski’s horses. Sci. Rep..

[295] Kartzinel TR (2015). DNA metabarcoding illuminates dietary niche partitioning by African large herbivores. Proc. Natl. Acad. Sci. U. S. A..

[296] Kelly EM, Sears KE (2011). Limb specialization in living marsupial and eutherian mammals: constraints on mammalian limb evolution. J. Mammal..

[297] Kelt DA, Meyer MD (2008). Body size frequency distributions in African mammals are bimodal at all spatial scales. Glob. Ecol. Biogeogr..

[298] Khadka KK, Singh N, Magar KT, James DA (2017). Dietary composition, breadth, and overlap between seasonally sympatric Himalayan musk deer and livestock: Conservation implications. Journal for Nature Conservation.

[299] Kim BJ, Lee NS, Lee SD (2011). Feeding diets of the Korean water deer (Hydropotes inermis argyropus) based on a 202 bp rbcL sequence analysis. Conservation Genetics.

[300] Kim DB, Koo KA, Kim HH, Hwang GY, Kong WS (2019). Reconstruction of the habitat range suitable for long-tailed goral (Naemorhedus caudatus) using fossils from the Paleolithic sites. Quaternary International.

[301] Koch, P. L. & Barnosky, A. D. Late Quaternary extinctions: state of the debate. *Annu. Rev. Ecol. Evol. Syst*. **37** (2006).

[302] Köhler M, Moyà-Solà S (2004). Reduction of brain and sense organs in the fossil insular bovid Myotragus. Brain. Behav. Evol..

[303] Kohn MJ, McKay MP (2012). Paleoecology of late Pleistocene–Holocene faunas of eastern and central Wyoming, USA, with implications for LGM climate models. Palaeogeography, Palaeoclimatology, Palaeoecology.

[304] Kohn MJ, McKay MP, Knight JL (2005). Dining in the Pleistocene—who’s on the menu?. Geology.

[305] Koike S, Nakashita R, Naganawa K, Koyama M, Tamura A (2013). Changes in diet of a small, isolated bear population over time. J. Mammal..

[306] Kosintsev P (2019). Evolution and extinction of the giant rhinoceros Elasmotherium sibiricum sheds light on late Quaternary megafaunal extinctions. Nature Ecology & Evolution.

[307] Kowalczyk R (2011). Influence of management practices on large herbivore diet—Case of European bison in Białowieża Primeval Forest (Poland). For. Ecol. Manage..

[308] Kram R, Dawson TJ (1998). Energetics and biomechanics of locomotion by red kangaroos (Macropus rufus). Comparative Biochemistry and Physiology Part B: Biochemistry and Molecular Biology.

[309] Krishna YC, Clyne PJ, Krishnaswamy J, Kumar NS (2009). Distributional and ecological review of the four horned antelope, Tetracerus quadricornis. Mammalia.

[310] Kropf M, Mead JI, Scott Anderson R (2007). Dung, diet, and the paleoenvironment of the extinct shrub-ox (Euceratherium Collinum) on the Colorado Plateau, USA. Quaternary Research.

[311] Kubo MO, Yamada E, Fujita M, Oshiro I (2015). Paleoecological reconstruction of Late Pleistocene deer from the Ryukyu Islands, Japan: Combined evidence of mesowear and stable isotope analyses. Palaeogeography, Palaeoclimatology, Palaeoecology.

[312] Kumar RS, Mishra C, Sinha A (2007). Foraging ecology and time-activity budget of the Arunachal macaque *Macaca munzala* – A preliminary study. Curr. Sci..

[313] Kuzmin YV (2010). Extinction of the woolly mammoth (Mammuthus primigenius) and woolly rhinoceros (Coelodonta antiquitatis) in Eurasia: review of chronological and environmental issues. Boreas.

[314] Lambert JE (1998). Primate digestion: interactions among anatomy, physiology, and feeding ecology. Evolutionary Anthropology.

[315] Lamoot I, Callebaut J, Demeulenaere E, Vandenberghe C, Hoffmann M (2005). Foraging behaviour of donkeys grazing in a coastal dune area in temperate climate conditions. Appl. Anim. Behav. Sci..

[316] Loponte DM, Corriale MJ (2013). Isotopic values of diet of Blastocerus dichotomus (marsh deer) in Paraná Basin, South America. Journal of Archaeological Science.

[317] Larramendi A (2015). Shoulder height, body mass, and shape of proboscideans. Acta Palaeontologica Polonica.

[318] Latham ADM (2020). A refined model of body mass and population density in flightless birds reconciles extreme bimodal population estimates for extinct moa. Ecography.

[319] Latrubesse EM (2010). The Late Miocene paleogeography of the Amazon Basin and the evolution of the Amazon River system. Earth-Science Reviews.

[320] Law A, Jones KC, Willby NJ (2014). Medium vs. short-term effects of herbivory by Eurasian beaver on aquatic vegetation. Aquat. Bot..

[321] Lazagabaster IA, Rowan J, Kamilar JM, Reed KE (2016). Evolution of craniodental correlates of diet in African Bovidae. J. Mamm. Evol..

[322] Lazagabaster IA (2018). Fossil Suidae (Mammalia, Artiodactyla) from Lee Adoyta, Ledi-Geraru, lower Awash Valley, Ethiopia: Implications for late Pliocene turnover and paleoecology. Palaeogeography, Palaeoclimatology, Palaeoecology.

[323] Lehmann, D. *Dietary and spatial strategies of gemsbok (Oryx g. gazella) and springbok (Antidorcas marsupialis) in response to drought in the desert environment of the Kunene region, Namibia* PhD thesis, Freie Universität Berlin (2015).

[324] Leslie, D. M. Boselaphus tragocamelus (Artiodactyla: Bovidae). *Mammalian Species*, 1–16 (2008).

[325] Leslie DM (2010). Procapra picticaudata (Artiodactyla: Bovidae). Mammalian Species.

[326] Leslie, D. M. & Schaller, G. B. Pantholops hodgsonii (Artiodactyla: Bovidae). *Mammalian Species*, 1–13 (2008).

[327] Leslie, D. M. & Schaller, G. B. Bos grunniens and Bos mutus (Artiodactyla: Bovidae). *Mammalian species*, 1–17 (2009).

[328] Leslie DM, Groves CP, Abramov AV (2010). Procapra przewalskii (Artiodactyla: Bovidae). Mammalian Species.

[329] Leslie DM, Lee DN, Dolman RW (2013). Elaphodus cephalophus (Artiodactyla: Cervidae). Mammalian Species.

[330] Leus K, Goodall GP, Macdonald AA (1999). Anatomy and histology of the babirusa (*Babyrousa babyrussa*) stomach. Comptes Rendus de l’Académie des Sciences - Series III - Sciences de la Vie.

[331] Li Y, Yu Y-Q, Shi L (2015). Foraging and bedding site selection by Asiatic ibex (Capra sibirica) during summer in Central Tianshan Mountains. Pakistan Journal of Zoology.

[332] Li, B., Xu, W., Blank, D. A., Wang, M. & Yang, W. Diet characteristics of wild sheep (Ovis ammon darwini) in the Mengluoke Mountains, Xinjiang. *China Journal of Arid Land* (2018).

[333] Liang X, Kang A, Pettorelli N (2017). Understanding habitat selection of the Vulnerable wild yak Bos mutus on the Tibetan Plateau. Oryx.

[334] Lister AM, Stuart AJ (2019). The extinction of the giant deer Megaloceros giganteus (Blumenbach): New radiocarbon evidence. Quaternary International.

[335] Liu X, Stanford CB, Yang J, Yao H, Li Y (2013). Foods Eaten by the Sichuan snub‐nosed monkey (Rhinopithecus roxellana) in Shennongjia National Nature Reserve, China, in relation to nutritional chemistry. Am. J. Primatol..

[336] Livezey BC (1993). An ecomorphological review of the dodo (Raphus cucullatus) and solitaire (Pezophaps solitaria), flightless Columbiformes of the Mascarene Islands. J. Zool..

[337] Livezey BC, Zusi RL (2007). Higher-order phylogeny of modern birds (Theropoda, Aves: Neornithes) based on comparative anatomy. II. Analysis and discussion. Zoological journal of the Linnean Society.

[338] Lobo, L. S. *Estudo da morfologia dentária de Xenorhinotherium bahiense Cartelle & Lessa, 1988 (Litopterna, Macraucheniidae)* Universidade Federal De Viçosa, (2015).

[339] Long, J. A., Archer, M., Flannery, T. & Hand, S. *Prehistoric mammals of Australia and New Guinea: one hundred million years of evolution*. (Johns Hopkins University Press, 2002).

[340] Louys J, Meloro C, Elton S, Ditchfield P, Bishop LC (2011). Mesowear as a means of determining diets in African antelopes. Journal of Archaeological Science.

[341] Ma J, Wang Y, Jin C, Hu Y, Bocherens H (2019). Ecological flexibility and differential survival of Pleistocene Stegodon orientalis and Elephas maximus in mainland southeast Asia revealed by stable isotope (C, O) analysis. Quaternary Science Reviews.

[342] MacFadden BJ (1986). Fossil horses from “Eohippus”(Hyracotherium) to Equus: scaling, Cope’s Law, and the evolution of body size. Paleobiology.

[343] MacFadden BJ (2005). Diet and habitat of toxodont megaherbivores (Mammalia, Notoungulata) from the late Quaternary of South and Central America. Quaternary Research.

[344] MacFadden BJ, Shockey BJ (1997). Ancient feeding ecology and niche differentiation of Pleistocene mammalian herbivores from Tarija, Bolivia: morphological and isotopic evidence. Paleobiology.

[345] MacPhee, R. D. E. & Sues, H.-D. *Extinctions in Near Time: Causes*, *Contexts, and Consequences*. (Springer, 1999).

[346] Madden, R. H. *Hypsodonty in Mammals: Evolution, Geomorphology, and the Role of Earth System Processes*. (Cambridge University Press, 2014).

[347] Al Majaini, H. Nutritional ecology of the Arabian tahr Hemitragus jayakari Thomas 1984 *in Wadi Sareen Reserve area*, M. Sc. thesis, Sultan Qaboos University, Oman. 97pages, (1999).

[348] Marcolino CP, dos Santos Isaias RM, Cozzuol MA, Cartelle C, Dantas MAT (2012). Diet of Palaeolama major (Camelidae) of Bahia, Brazil, inferred from coprolites. Quaternary international.

[349] Marin VC (2020). Diet of the marsh deer in the Paraná River Delta, Argentina—a vulnerable species in an intensive forestry landscape. European Journal of Wildlife Research.

[350] Marinero NV, Navarro JL, Martella MB (2017). Does food abundance determine the diet of the Puna Rhea (Rhea tarapacensis) in the Austral Puna desert in Argentina?. Emu-Austral Ornithology.

[351] Mayte G-B (2016). Diet and habitat of Mammuthus columbi (Falconer, 1857) from two Late Pleistocene localities in central western Mexico. Quaternary International.

[352] McAfee RK (2011). Feeding mechanics and dietary implications in the fossil sloth Neocnus (Mammalia: Xenarthra: Megalonychidae) from Haiti. J. Morphol..

[353] McDonald HG (2005). Palecology of extinct Xenarthrans and the Great American Biotic Interchange. Bulletin of the Florida Museum of Natural History.

[354] McDonald HG, Pelikan S (2006). Mammoths and mylodonts: Exotic species from two different continents in North American Pleistocene faunas. Quaternary International.

[355] McDonald HG, Feranec RS, Miller N (2019). First record of the extinct ground sloth, Megalonyx jeffersonii,(Xenarthra, Megalonychidae) from New York and contributions to its paleoecology. Quaternary International.

[356] McFarlane DA, MacPhee RDE, Ford DC (1998). Body Size Variability and a Sangamonian Extinction Model forAmblyrhiza, a West Indian Megafaunal Rodent. Quaternary Research.

[357] McNamara, K. & Murray, P. *Prehistoric Mammals of Western Australia*. (Western Australian Museum, 2010).

[358] Mead JI, O’Rourke MK, Foppe TM (1986). Dung and diet of the extinct Harrington’s mountain goat (*Oreamnos harringtoni*). J. Mammal..

[359] Mead JI, Agenbroad LD, Phillips AM, Middleton LT (1987). Extinct mountain goat (Oreamnos harringtoni) in southeastern Utah. Quaternary Research.

[360] Meijaard E, Groves C (2002). Upgrading three subspecies of babirusa (Babyrousa sp.) to full species level. Asian Wild Pig News.

[361] Meijaard E, Groves CP (2004). Morphometrical relationships between South‐east Asian deer (Cervidae, tribe Cervini): Evolutionary and biogeographic implications. J. Zool..

[362] Meloro C, de Oliveira AM (2019). Elbow joint geometry in bears (Ursidae, Carnivora): a tool to infer paleobiology and functional adaptations of Quaternary fossils. J. Mamm. Evol..

[363] Mengli Z, Willms WD, Guodong H, Ye J (2006). Bactrian camel foraging behaviour in a Haloxylon ammodendron (C.A. Mey) desert of Inner Mongolia. Appl. Anim. Behav. Sci..

[364] Miller GH (1999). Pleistocene extinction of Genyornis newtoni: human impact on Australian megafauna. Science.

[365] Miller GH (2005). Ecosystem collapse in Pleistocene Australia and a human role in megafaunal extinction. Science.

[366] Milligan HE, Humphries MM (2010). The importance of aquatic vegetation in beaver diets and the seasonal and habitat specificity of aquatic-terrestrial ecosystem linkages in a subarctic environment. Oikos.

[367] Milton, S. J., Dean, W. R. J. & Siegfried, W. R. Food selection by ostrich in southern Africa. *The Journal of wildlife management*, 234–248 (1994).

[368] Mimoun JB, Nouira S (2015). Food habits of the aoudad Ammotragus lervia in the Bou Hedma mountains, Tunisia. South African Journal of Science.

[369] Mingxing D, Yanhong Z, Jianguo Z (2014). Cold and/or wet Early Holocene in Shijiazhuang district: Evidences from tooth microwear and stable isotopes analyses. Quaternary Sciences.

[370] Miranda M (2012). Contrasting feeding patterns of native red deer and two exotic ungulates in a Mediterranean ecosystem. Wildlife Research.

[371] Missagia RV, Parisi-Dutra R, Cozzuol MA (2016). Morphometry of Catagonus stenocephalus (Lund in Reinhardt 1880)(Artiodactyla: Tayassuidae) and taxonomical considerations about Catagonus Ameghino 1904. Lundiana International Journal of Biodiversity.

[372] Mitchell DR, Wroe S (2019). Biting mechanics determines craniofacial morphology among extant diprotodont herbivores: dietary predictions for the giant extinct short-faced kangaroo, Simosthenurus occidentalis. Paleobiology.

[373] Mitchell KJ (2014). Ancient DNA reveals elephant birds and kiwi are sister taxa and clarifies ratite bird evolution. Science.

[374] Moczygemba, J. D. *Movements of nilgai antelope (Boselaphus tragocamelus) in southern Texas*. (Texas A&M University-Kingsville, 2010).

[375] Moore DM (1978). Post-glacial vegetation in the South Patagonian territory of the giant ground sloth, Mylodon. Botanical Journal of the Linnean Society.

[376] Mori E, Bozzi R, Laurenzi A (2017). Feeding habits of the crested porcupine Hystrix cristata L. 1758 (Mammalia, Rodentia) in a Mediterranean area of Central Italy. The European Zoological Journal.

[377] Morosi, E. & Ubilla, M. Dietary and palaeoenvironmental inferences in Neolicaphrium recens Frenguelli, 1921 (Litopterna, Proterotheriidae) using carbon and oxygen stable isotopes (Late Pleistocene; Uruguay). *Hist. Biol.* 1–7, 10.1080/08912963.2017.1355914 (2017).

[378] Murray, P. F. & Vickers-Rich, P. *Magnificent mihirungs: the colossal flightless birds of the Australian dreamtime*. (Indiana University Press, 2004).

[379] Naish, D. *The anatomy of sloths*, https://blogs.scientificamerican.com/tetrapod-zoology/the-anatomy-of-sloths/ (2012).

[380] Nedin C (1991). The dietary niche of the extinct Australian marsupial lion: Thylacoleo carnifex Owen. Lethaia.

[381] New Zealand Organisms Register. (New Zealand, 2020).

[382] Nijboer, J. & Clauss, M. *Fibre intake and feces quality in leaf-eating primates* PhD thesis, Utrecht University, (2006).

[383] Noe-Nygaard N, Price TD, Hede SU (2005). Diet of aurochs and early cattle in southern Scandinavia: evidence from 15N and 13C stable isotopes. Journal of Archaeological Science.

[384] Northcote EM (1982). Size, form and habit of the extinct Maltese swan Cygnus falconeri. Ibis.

[385] Nowak, R. M. *Walker’s Mammals of the World*. (Johns Hopkins University Press, 1999).

[386] Nugraha R, Mustari AH (2017). Habitat Characteristics and Diet of Bear Cuscus (Ailurops ursinus) in Tanjung Peropa Wildlife Reserve, Southeast Sulawesi. Jurnal Wasian.

[387] Oli CB (2018). Dry season diet composition of four-horned antelope Tetracerus quadricornis in tropical dry deciduous forests, Nepal. PeerJ.

[388] de Oliveira, J. F., Asevedo, L., Cherkinsky, A. & Dantas, M. A. T. Radiocarbon dating and integrative paleoecology (δ13C, stereomicrowear) of Eremotherium laurillardi (LUND, 1842) from midwest region of the Brazilian intertropical region. *Journal of South American Earth Sciences*, 102653 (2020).

[389] Olson VA, Turvey ST (2013). The evolution of sexual dimorphism in New Zealand giant moa (Dinornis) and other ratites. Proc. R. Soc. B..

[390] Omena, É. C., Silva, J. L. L. d., Sial, A. N., Cherkinsky, A. & Dantas, M. A. T. Late Pleistocene meso-megaherbivores from Brazilian Intertropical Region: isotopic diet (δ 13C), niche differentiation, guilds and paleoenvironmental reconstruction (δ 13C, δ 18O). *Hist. Biol*., 1–6 (2020).

[391] Osawa R (1990). Feeding strategies of the swamp wallaby, Wallabia bicolor, on North Stradbroke Island, Queensland. I: Composition of diets. Wildlife Research.

[392] Pacini, N. & Harper, D. M. in *Tropical stream ecology* 147–197 (Elsevier, 2008).

[393] The Paleobiology Database. (University of Wisconsin-Madison, Department of Geosciences 2020).

[394] Palmqvist P, Martínez-Navarro B, Arribas A (1996). Prey selection by terrestrial carnivores in a lower Pleistocene paleocommunity. Paleobiology.

[395] Palmqvist P, Gröcke DR, Arribas A, Fariña RA (2003). Paleoecological reconstruction of a lower Pleistocene large mammal community using biogeochemical (δ13C, δ15N, δ18O, Sr: Zn) and ecomorphological approaches. Paleobiology.

[396] Palmqvist P, Pérez-Claros JA, Janis CM, Gröcke DR (2008). Tracing the ecophysiology of ungulates and predator–prey relationships in an early Pleistocene large mammal community. Palaeogeography, Palaeoclimatology, Palaeoecology.

[397] Palombo, M. R. in *Insular Vertebrate Evolution: the Palaeontological Approach* Vol. 12 (eds Josep Antoni Alcover & P. Bover) 233–244 (Monografies de la Societat d’História Natural de les Balears, 2005).

[398] Palombo MR (2005). Coupling tooth microwear and stable isotope analyses for palaeodiet reconstruction: the case study of Late Middle Pleistocene Elephas (Palaeoloxodon) antiquus teeth from Central Italy (Rome area). Quaternary International.

[399] Pangau-Adam M, Muehlenberg M (2014). Palm species in the diet of the northern cassowary (Casuarius unappendiculatus) in Jayapura region, Papua, Indonesia. Palms.

[400] Pansani TR, Muniz FP, Cherkinsky A, Pacheco MLAF, Dantas MAT (2019). Isotopic paleoecology (δ13C, δ18O) of Late Quaternary megafauna from Mato Grosso do Sul and Bahia States, Brazil. Quaternary Science Reviews.

[401] Pansu J (2019). Trophic ecology of large herbivores in a reassembling African ecosystem. J. Ecol..

[402] Paoletti G, Puig S (2007). Diet of the Lesser Rhea (Pterocnemia pennata) and availability of food in the Andean Precordillera (Mendoza, Argentina). Emu-Austral Ornithology.

[403] Pappa S, Schreve DC, Rivals F (2019). The bear necessities: A new dental microwear database for the interpretation of palaeodiet in fossil Ursidae. Palaeogeography, Palaeoclimatology, Palaeoecology.

[404] Park J-E, Kim B-J, Oh D-H, Lee H, Lee S-D (2011). Feeding habit analysis of the Korean water deer. Korean Journal of Environment and Ecology.

[405] Patnaik R (2015). Diet and habitat changes among Siwalik herbivorous mammals in response to Neogene and Quaternary climate changes: An appraisal in the light of new data. Quaternary International.

[406] Patnaik R, Singh NP, Paul D, Sukumar R (2019). Dietary and habitat shifts in relation to climate of Neogene-Quaternary proboscideans and associated mammals of the Indian subcontinent. Quaternary Science Reviews.

[407] Peigné S (2009). Predormancy omnivory in European cave bears evidenced by a dental microwear analysis of Ursus spelaeus from Goyet, Belgium. Proc. Natl. Acad. Sci. USA.

[408] Pereira JA, Quintana RD, Monge S (2003). Diets of plains vizcacha, greater rhea and cattle in Argentina. Rangeland Ecology & Management/Journal of Range Management Archives.

[409] Pereira ICdS, Dantas MAT, Ferreira RL (2013). Record of the giant sloth *Valgipes bucklandi* (Lund, 1839) (Tardigrada, Scelidotheriinae) in Rio Grande do Norte state, Brazil, with notes on taphonomy and paleoecology. Journal of South American Earth Sciences.

[410] Pérez-Crespo VA (2012). Geographic variation of diet and habitat of the Mexican populations of Columbian Mammoth (Mammuthus columbi). Quaternary International.

[411] Pérez ME, Vallejo-Pareja MC, Carrillo JD, Jaramillo C (2017). A new Pliocene capybara (Rodentia, Caviidae) from Northern South America (Guajira, Colombia), and its implications for the great American biotic interchange. J. Mamm. Evol..

[412] Pérez-Crespo VA, Arroyo-Cabrales J, Alva-Valdivia LM, Morales-Puente P, Cienfuegos-Alvarado E (2011). Diet and habitat definitions for Mexican glyptodonts from Cedral (San Luis Potosí, México) based on stable isotope analysis. Geol. Mag..

[413] Pérez-Crespo VA (2020). Isotopic paleoecology of a toxodont Mixotoxodon larensis from Michoacan, Mexico. The Southwestern Naturalist.

[414] Phillips MJ, Gibb GC, Crimp EA, Penny D (2010). Tinamous and moa flock together: mitochondrial genome sequence analysis reveals independent losses of flight among ratites. Syst. Biol..

[415] Pinto-Llona AC (2013). Macrowear and occlusal microwear on teeth of cave bears Ursus spelaeus and brown bears Ursus arctos: Inferences concerning diet. Palaeogeography, Palaeoclimatology, Palaeoecology.

[416] Plint T, Longstaffe FJ, Zazula G (2019). Giant beaver palaeoecology inferred from stable isotopes. Sci. Rep..

[417] Poinar HN (1998). Molecular coproscopy: dung and diet of the extinct ground sloth Nothrotheriops shastensis. Science.

[418] Pokharel KP, Yohannes E, Salvarina I, Storch I (2015). Isotopic evidence for dietary niche overlap between barking deer and four-horned antelope in Nepal. Journal of Biological Research-Thessaloniki.

[419] Pokhrel K, Poudel P, Neupane B, Paudel R (2019). Comparative study in habitat suitability analysis of wild water buffalo (Bubalus arnee) in two flood plains of Chitwan National Park (CNP). Nepal. International Journal of Research Studies in Zoology.

[420] Poole KG, Heard DC (2003). Seasonal habitat use and movements of mountain goats, Oreamnos americanus, in east-central British Columbia. The Canadian Field-Naturalist.

[421] Presslee S (2019). Palaeoproteomics resolves sloth relationships. Nature Ecology & Evolution.

[422] Prevosti FJ, Martin FM (2013). Paleoecology of the mammalian predator guild of Southern Patagonia during the latest Pleistocene: ecomorphology, stable isotopes, and taphonomy. Quaternary International.

[423] Prevosti, F. J. & Vizcaíno, S. F. Paleoecology of the large carnivore guild from the late Pleistocene of Argentina. *Acta Palaeontologica Polonica* **51** (2006).

[424] Price, G. J. *et al*. Seasonal migration of marsupial megafauna in Pleistocene Sahul (Australia-New Guinea). *Proc. Biol. Sci*. **284**, 10.1098/rspb.2017.0785 (2017). 10.1098/rspb.2017.0785 PMC5627191 28954903

[425] Prideaux GJ (1999). Borungaboodie hatcheri gen. et sp. nov., a very large bettong (Marsupialia: Macropodoidea) from the Pleistocene of southwestern Australia. Records of the Western Australian Museum.

[426] Prideaux GJ (2007). An arid-adapted middle Pleistocene vertebrate fauna from south-central Australia. Nature.

[427] Prideaux GJ (2009). Extinction implications of a chenopod browse diet for a giant Pleistocene kangaroo. Proc. Natl. Acad. Sci. USA.

[428] Prideaux GJ (2010). Timing and dynamics of Late Pleistocene mammal extinctions in southwestern Australia. Proc. Natl. Acad. Sci. USA.

[429] Prothero, D. R. *Giants of the Lost World: Dinosaurs and Other Extinct Monsters of South America*. (Smithsonian Institution, 2016).

[430] Pujaningsih R (2009). Diet composition of Anoa (Buballus sp.) studied using direct observation and dung analysis method in their habitat. Journal of the Indonesian Tropical Animal Agriculture.

[431] Pujos F, Argot C, De Iuliis G, Werdelin L (2007). A peculiar climbing Megalonychidae from the Pleistocene of Peru and its implication for sloth history. Zoological Journal of the Linnean Society.

[432] Pujos F, Gaudin TJ, De Iuliis G, Cartelle C (2012). Recent Advances on Variability, Morpho-Functional Adaptations, Dental Terminology, and Evolution of Sloths. J. Mamm. Evol..

[433] Pushkina D, Bocherens H, Ziegler R (2014). Unexpected palaeoecological features of the Middle and Late Pleistocene large herbivores in southwestern Germany revealed by stable isotopic abundances in tooth enamel. Quaternary International.

[434] Puspaningrum, M. R. *et al*. in *VIth International Conference on Mammoths and their Relatives* Vol. 102 (School of Geology, Aristotle University of Thessaloniki, Greece: Aristotle University of Thessaloniki, 2014).

[435] Puspaningrum MR, van den Bergh GD, Chivas AR, Setiabudi E, Kurniawan I (2020). Isotopic reconstruction of Proboscidean habitats and diets on Java since the Early Pleistocene: Implications for adaptation and extinction. Quaternary Science Reviews.

[436] Quin B (1996). Diet and habitat of emus Dromaius novaehollandiae in the Grampians Ranges, south-western Victoria. Emu.

[437] Rahman MM (2015). Feeding ecology of Northern Plains Sacred langur Semnopithecus entellus (Dufresne) in Jessore, Bangladesh: dietary composition, seasonal and age-sex differences. Asian Primates J.

[438] Raia P, Carotenuto F, Meiri S (2010). One size does not fit all: no evidence for an optimal body size on islands. Glob. Ecol. Biogeogr..

[439] Rayé G (2011). New insights on diet variability revealed by DNA barcoding and high-throughput pyrosequencing: chamois diet in autumn as a case study. Ecol. Res..

[440] Rduch V (2016). Diet of the puku antelope (Kobus vardonii) and dietary overlap with selected other bovids in Kasanka National Park, Zambia. Mammal Research.

[441] Reid, F. A. *A Field Guide to the Mammals of Central America & Southeast Mexico*. (Oxford University Press, 2009).

[442] Remington TE (1989). Why do grouse have ceca? A test of the fiber digestion theory. J. Exp. Zool..

[443] Repi, T., Masyud, B., Mustari, A. H. & Prasetyo, L. B. Daily activity and diet of Talaud bear cuscus (Ailurops melanotis Thomas, 1898) on Salibabu Island, North Sulawesi, Indonesia. *Biodiversitas Journal of Biological Diversity* **20** (2019).

[444] Resar, N. A. *Reconstructing the Paleodiet of Ground Sloths Using Microwear Analysis*, Kent State University, (2012).

[445] Resar NA, Green JL, McAfee RK (2013). Reconstructing paleodiet in ground sloths (Mammalia, Xenarthra) using dental microwear analysis. Kirtlandia.

[446] Reus ML (2014). Trophic interactions between the native guanaco (Lama guanicoe) and the exotic donkey (Equus asinus) in the hyper-arid Monte desert (Ischigualasto Park, Argentina). Stud. Neotrop. Fauna Environ..

[447] Reynolds PS (2002). How big is a giant? The importance of method in estimating body size of extinct mammals. J. Mammal..

[448] Richards MP (2008). Isotopic evidence for omnivory among European cave bears: Late Pleistocene Ursus spelaeus from the Peştera cu Oase. Romania. Proc. Natl. Acad. Sci. USA.

[449] Richards HL, Wells RT, Evans AR, Fitzgerald EMG, Adams JW (2019). The extraordinary osteology and functional morphology of the limbs in Palorchestidae, a family of strange extinct marsupial giants. PLoS One.

[450] Righini, N. & Amato, K. R. in *Encyclopedia of Animal Cognition and Behavior* 1–6 (Springer, Cham, 2018).

[451] Rijsdijk KF (2009). Mid-Holocene vertebrate bone Concentration-Lagerstätte on oceanic island Mauritius provides a window into the ecosystem of the dodo (Raphus cucullatus). Quaternary Science Reviews.

[452] Rishworth C, McIlroy J, Tanton M (1995). Diet of the common wombat, Vombatus ursinus, in plantations of Pinus radiata. Wildlife Research.

[453] Rivals, F. Les petits bovidés (Caprini et Rupicaprini) pléistocènes dans le bassin méditerranéen et le Caucase. (Archaeopress, Oxford, England, 2004).

[454] Rivals F, Lister AM (2016). Dietary flexibility and niche partitioning of large herbivores through the Pleistocene of Britain. Quaternary Science Reviews.

[455] Rivals F, Schulz E, Kaiser TM (2009). Late and middle Pleistocene ungulates dietary diversity in Western Europe indicate variations of Neanderthal paleoenvironments through time and space. Quaternary Science Reviews.

[456] Rivals F, Semprebon G, Lister A (2012). An examination of dietary diversity patterns in Pleistocene proboscideans (Mammuthus, Palaeoloxodon, and Mammut) from Europe and North America as revealed by dental microwear. Quaternary International.

[457] Rivals F, Rindel D, Belardi JB (2013). Dietary ecology of extant guanaco (Lama guanicoe) from Southern Patagonia: seasonal leaf browsing and its archaeological implications. Journal of Archaeological Science.

[458] Rivals F, Takatsuki S, Albert RM, Macià L (2014). Bamboo feeding and tooth wear of three sika deer (Cervus nippon) populations from northern Japan. J. Mammal..

[459] Rivals F, Sanz M, Daura J (2016). First reconstruction of the dietary traits of the Mediterranean deer (Haploidoceros mediterraneus) from the Cova del Rinoceront (NE Iberian Peninsula). Palaeogeography, Palaeoclimatology, Palaeoecology.

[460] Rivals F, Semprebon GM, Lister AM (2019). Feeding traits and dietary variation in Pleistocene proboscideans: A tooth microwear review. Quaternary Science Reviews.

[461] Robinson, A. C. & Young, M. C. The Toolache wallaby (Macropus greyi waterhouse). (Department of Environment and Planning, South Australian National Parks and Wildlife Service, 1983).

[462] Robu M (2013). Isotopic evidence for dietary flexibility among European Late Pleistocene cave bears (Ursus spelaeus). Can. J. Zool..

[463] Ross S, Al Jahdhami MH, Al Rawahi H (2019). Refining conservation strategies using distribution modelling: a case study of the Endangered Arabian tahr Arabitragus jayakari. Oryx.

[464] Rotti A, Mothé D, dos Santos Avilla L, Semprebon GM (2018). Diet reconstruction for an extinct deer (Cervidae: Cetartiodactyla) from the Quaternary of South America. Palaeogeography, Palaeoclimatology, Palaeoecology.

[465] Rowan J, Faith JT, Gebru Y, Fleagle JG (2015). Taxonomy and paleoecology of fossil Bovidae (Mammalia, Artiodactyla) from the Kibish Formation, southern Ethiopia: Implications for dietary change, biogeography, and the structure of the living bovid faunas of East Africa. Palaeogeography, Palaeoclimatology, Palaeoecology.

[466] Rowan J, Martini P, Likius A, Merceron G, Boisserie J-R (2019). New Pliocene remains of *Camelus grattardi* (Mammalia, Camelidae) from the Shungura Formation, Lower Omo Valley, Ethiopia, and the evolution of African camels. Hist. Biol..

[467] Roy D, Ashokkumar M, Desai AA (2012). Foraging ecology of Nilgiri Langur (Trachypithecus johnii) in Parimbikulam Tiger Reserve, Kerala, India. Asian Journal of Conservation Biology.

[468] Rozzi R (2017). A new extinct dwarfed buffalo from Sulawesi and the evolution of the subgenus Anoa: An interdisciplinary perspective. Quaternary Science Reviews.

[469] Ruez DR (2005). Diet of Pleistocene *Paramylodon harlani* (Xenarthra: Mylodontidae): review of methods and preliminary use of carbon isotopes. Texas Journal of Science.

[470] Ruso GE (2017). Beatragus hunteri (Artiodactyla: Bovidae). Mammalian Species.

[471] Rybczynski N (2007). Castorid phylogenetics: implications for the evolution of swimming and tree-exploitation in beavers. J. Mamm. Evol..

[472] Saarinen J, Karme A (2017). Tooth wear and diets of extant and fossil xenarthrans (Mammalia, Xenarthra) – Applying a new mesowear approach. Palaeogeography, Palaeoclimatology, Palaeoecology.

[473] Saarinen J, Eronen J, Fortelius M, Seppä H, Lister AM (2016). Patterns of diet and body mass of large ungulates from the Pleistocene of Western Europe, and their relation to vegetation. Palaeontologia Electronica.

[474] Salas LA, Fuller TK (1996). Diet of the lowland tapir (Tapirus terrestris L.) in the Tabaro River valley, southern Venezuela. Can. J. Zool..

[475] Sales J (2006). Digestive physiology and nutrition of ratites. Avian and poultry biology reviews.

[476] Salesa MJ (2006). Anatomy of the “false thumb” of Tremarctos ornatus (Carnivora, Ursidae, Tremarctinae): phylogenetic and functional implications. Estudios Geologicos.

[477] Salles LO (2016). A new record of a Scelidotheriine ground sloth (Xenarthra, Mylodontidae) from Central Brazil: Quaternary cave stratigraphy, taxonomy and stable isotopes. Palaeogeography, Palaeoclimatology, Palaeoecology.

[478] San Diego Zoo Global. *San Diego (CA): Tapirs (extant/living species; Tapirus spp.) Fact Sheet*, http://ielc.libguides.com/sdzg/factsheets/tapirs (c2009-2019).

[479] Sánchez B, Prado JL, Alberdi MT (2004). Feeding ecology, dispersal, and extinction of South American Pleistocene gomphotheres (Gomphotheriidae, Proboscidea). Paleobiology.

[480] Sánchez B, Prado JL, Alberdi MT (2006). Ancient feeding, ecology and extinction of Pleistocene horses from the Pampean Region, Argentina. Ameghiniana.

[481] Sankar K (2013). Home range, habitat use and food habits of re-introduced gaur (Bos gaurus gaurus) in Bandhavgarh Tiger Reserve, central India. Tropical Conservation Science.

[482] Sarhangzadeh J, Yavari A, Hemami M, Jafari H, Shams-Esfandabad B (2013). Habitat suitability modeling for wild goat (Capra aegagrus) in a mountainous arid area, central Iran. Caspian Journal of Environmental Sciences.

[483] Scasta JD, Beck JL, Angwin CJ (2016). Meta-Analysis of Diet Composition and Potential Conflict of Wild Horses with Livestock and Wild Ungulates on Western Rangelands of North America. Rangeland Ecology & Management.

[484] Schaller GB, Khan SA (1975). Distribution and status of markhor (Capra falconeri. Biol. Conserv..

[485] Schaller G (1986). Feeding behavior of Sichuan takin (Budorcas taxicolor). Mammalia.

[486] Schilling, A.-M. & Rössner, G. E. The (sleeping) Beauty in the Beast–a review on the water deer, Hydropotes inermis. *Hystrix, the Italian Journal of Mammalogy* **28** (2017).

[487] Schmidt CW (2008). Dental microwear analysis of extinct flat-headed peccary (*Platygonus compressus*) from Southern Indiana. Proc. Indiana Acad. Sci..

[488] Schubert BW, Graham RW, McDonald HG, Grimm EC, Stafford TW (2004). Latest Pleistocene paleoecology of Jefferson’s ground sloth (Megalonyx jeffersonii) and elk-moose (Cervalces scotti) in northern Illinois. Quaternary Research.

[489] Schulz E (2013). Food preferences and tooth wear in the sand gazelle (Gazella marica). Mammalian Biology.

[490] Seegmiller RF, Ohmart RD (1981). Ecological relationships of feral burros and desert bighorn sheep. Wildlife Monographs.

[491] Semprebon GM, Rivals F (2010). Trends in the paleodietary habits of fossil camels from the Tertiary and Quaternary of North America. Palaeogeography, Palaeoclimatology, Palaeoecology.

[492] Semprebon GM (2015). Dietary reconstruction of pygmy mammoths from Santa Rosa Island of California. Quaternary International.

[493] Serbent M, Periago ME, Leynaud GC (2011). Mazama gouazoubira (Cervidae) diet during the dry season in the arid Chaco of Córdoba (Argentina). J. Arid Environ..

[494] Severud WJ, Belant JL, Windels SK, Bruggink JG (2013). Seasonal variation in assimilated diets of American beavers. The American Midland Naturalist.

[495] Sewell L, Merceron G, Hopley PJ, Zipfel B, Reynolds SC (2019). Using springbok (Antidorcas) dietary proxies to reconstruct inferred palaeovegetational changes over 2 million years in Southern Africa. Journal of Archaeological Science: Reports.

[496] Shapiro LJ (2005). Morphometric analysis of lumbar vertebrae in extinct Malagasy strepsirrhines. *Am*. J. Phys. Anthropol..

[497] Sharp AC, Rich TH (2016). Cranial biomechanics, bite force and function of the endocranial sinuses in Diprotodon optatum, the largest known marsupial. J. Anat..

[498] Shockey BJ (2001). Specialized knee joints in some extinct, endemic, South American herbivores. Acta Palaeontologica Polonica.

[499] Shrestha, T. K., Hecker, L. J., Aryal, A. & Coogan, S. C. Feeding preferences and nutritional niche of wild water buffalo (Bubalus arnee) in Koshi Tappu Wildlife Reserve, Nepal. *Ecol. Evol*. (2020). 10.1002/ece3.6183 PMC7391305 32760500

[500] Simpson, B. K., Shukor, M. & Magintan, D. in *AIP Conference Proceedings*. 317–324 (American Institute of Physics).

[501] Smith GJ, DeSantis LR (2018). Dietary ecology of Pleistocene mammoths and mastodons as inferred from dental microwear textures. Palaeogeography, Palaeoclimatology, Palaeoecology.

[502] Smith RJ, Jungers WL (1997). Body mass in comparative primatology. J. Hum. Evol..

[503] Smith, A. T. *et al*. *A Guide to the Mammals of China*. (Princeton University Press, 2008).

[504] Smith FA, Elliott SM, Lyons SK (2010). Methane emissions from extinct megafauna. Nature Geoscience.

[505] Soibelzon, L. H. L Ursidae (Carnivora, Fissipedia) fósiles de la República Argentina. *Aspectos Sistemáticos y Paleoecológicos*. (2002).

[506] Sondaar, P. Y. & van der Geer, S. A. in *Archaeozoology of the Near East IVA*. *Proceedings of the fourth international symposium on the archaeozoology of Southwestern Asia and adjacent areas (ARC Publicatie 32*, pp. 67–73). *Groningen*: Centrum voor Archeologische Research & Consultancy.

[507] Sony R, Sen S, Kumar S, Sen M, Jayahari K (2018). Niche models inform the effects of climate change on the endangered Nilgiri Tahr (Nilgiritragus hylocrius) populations in the southern Western Ghats, India. Ecol. Eng..

[508] Spitzer, R. *et al*. Fifty years of European ungulate dietary studies: a synthesis. *Oikos***n/a**, 10.1111/oik.07435 (2020).

[509] Squires, J. R. & Anderson, S. H. Trumpeter swan (Cygnus buccinator) food habits in the Greater Yellowstone Ecosystem. *American Midland Naturalist*, 274–282 (1995).

[510] St-Louis, A. & Côté, S. D. Equus kiang (Perissodactyla: Equidae). *Mammalian Species*, 1–11 (2009).

[511] Steenweg R, Hebblewhite M, Gummer D, Low B, Hunt B (2016). Assessing potential habitat and carrying capacity for reintroduction of plains bison (Bison bison bison) in Banff National Park. PLoS One.

[512] Stefaniak, K. *et al*. Browsers, grazers or mix-feeders? Study of the diet of extinct Pleistocene Eurasian forest rhinoceros Stephanorhinus kirchbergensis (Jäger, 1839) and woolly rhinoceros Coelodonta antiquitatis (Blumenbach, 1799). *Quaternary International* (2020).

[513] Steinmetz RG (2004). Bos gaurus) and banteng (B. javanicus) in the lowland forest mosaic of Xe Pian Protected Area, Lao PDR: abundance, habitat use, and conservation. Mammalia.

[514] Stinnesbeck SR (2017). A new fossil peccary from the Pleistocene-Holocene boundary of the eastern Yucatán Peninsula, Mexico. Journal of South American Earth Sciences.

[515] Stirrat SC (2002). Foraging ecology of the agile wallaby (Macropus agilis) in the wet–dry tropics. Wildlife Research.

[516] Stuart AJ (1991). Mammalian extinctions in the Late Pleistocene of northern Eurasia and North America. Biological Reviews.

[517] Stuenes S (1989). Taxonomy, habits, and relationships of the subfossil Madagascan hippopotami Hippopotamus lemerlei and H. madagascariensis. Journal of Vertebrate Paleontology.

[518] Burnik Šturm M, Ganbaatar O, Voigt CC, Kaczensky P (2017). Sequential stable isotope analysis reveals differences in dietary history of three sympatric equid species in the Mongolian Gobi. J. Appl. Ecol..

[519] Stynder DD (2009). The diets of ungulates from the hominid fossil-bearing site of Elandsfontein, Western Cape, South Africa. Quaternary Research.

[520] Sugimoto T (2018). Diet of sympatric wild and domestic ungulates in southern Mongolia by DNA barcoding analysis. J. Mammal..

[521] Superina M, Loughry W (2012). Life on the half-shell: consequences of a carapace in the evolution of armadillos (Xenarthra: Cingulata). J. Mamm. Evol..

[522] Syed Z, Ilyas O (2016). Habitat preference and feeding ecology of alpine musk deer (Moschus chrysogaster) in Kedarnath Wildlife Sanctuary, Uttarakhand, India. Animal Production Science.

[523] Takada H, Minami M (2019). Food habits of the Japanese serow (Capricornis crispus) in an alpine habitat on Mount Asama, central Japan. Mammalia.

[524] Takada H, Nakamura K, Minami M (2019). Effects of the physical and social environment on flight response and habitat use in a solitary ungulate, the Japanese serow (Capricornis crispus). Behav. Processes.

[525] Takai M (2011). Stable isotope analysis of the tooth enamel of Chaingzauk mammalian fauna (late Neogene, Myanmar) and its implication to paleoenvironment and paleogeography. Palaeogeography, Palaeoclimatology, Palaeoecology.

[526] Talamoni SA, Assis MA (2009). Feeding habit of the Brazilian tapir, Tapirus terrestris (Perissodactyla: Tapiridae) in a vegetation transition zone in south-eastern Brazil. Zoologia (Curitiba).

[527] Talbot LM, Talbot MH (1965). The tamarau (*Bubalus mindorensis* (Huede)) observations and recommendations. Mammalia.

[528] Teague, R. L. *The ecological context of the Early Pleistocene hominin dispersal to Asia* PhD thesis, The George Washington University, (2001).

[529] Teale CL, Miller NG (2012). Mastodon herbivory in mid-latitude late-Pleistocene boreal forests of eastern North America. Quaternary Research.

[530] Telfer WR, Bowman DMJS (2006). Diet of four rock-dwelling macropods in the Australian monsoon tropics. Austral Ecol..

[531] Tewari R, Rawat G (2013). Studies on the Food and Feeding Habits of Swamp Deer (Rucervus duvaucelii duvaucelii) in Jhilmil Jheel Conservation Reserve, Haridwar, Uttarakhand, India. International Scholarly Research Notices.

[532] Thuc, P. D., Hieu, D. N., Thap, H. V., Van, V. H. & Khu, N. X. Notes on food of Capricornis milneedwardsii in the Cat Ba archipelago, Hai Phong, Vietnam. *TAP CHI SINH HOC (Journal of Biology)* **34** (2012).

[533] Thuc PD, Baxter G, Smith C, Hieu DN (2014). Population status of the Southwest China serow Capricornis milneedwardsii: a case study in Cat Ba Archipelago, Vietnam. Pac. Conserv. Biol..

[534] Tiunov AV, Kirillova IV (2010). Stable isotope (13C/12C and 15N/14N) composition of the woolly rhinoceros Coelodonta antiquitatis horn suggests seasonal changes in the diet. Rapid Commun. Mass Spectrom..

[535] Tobler MW (2002). Habitat use and diet of Baird’s Tapirs (Tapirus bairdii) in a montane cloud forest of the Cordillera de Talamanca, Costa Rica. Biotropica.

[536] Tobler, M., Naranjo, E. J. & Lira-Torres, I. in *Ecology and conservation of Neotropical montane oak forests* 347–359 (Springer, 2006).

[537] Tochigi K (2018). Detection of arboreal feeding signs by Asiatic black bears: effects of hard mast production at individual tree and regional scales. J. Zool..

[538] Tonni, E. Los mamíferos del Cuaternario de la región pampeana de Buenos Aires, Argentina. (2009).

[539] Torres MM, Puig S (2010). Seasonal diet of vicuñas in the Los Andes protected area (Salta, Argentina): Are they optimal foragers?. J. Arid Environ..

[540] Torres CR, Clarke JA (2018). Nocturnal giants: evolution of the sensory ecology in elephant birds and other palaeognaths inferred from digital brain reconstructions. Proceedings of the Royal Society B.

[541] Tovondrafale T, Razakamanana T, Hiroko K, Rasoamiaramanana A (2014). Paleoecological analysis of elephant bird (Aepyornithidae) remains from the Late Pleistocene and Holocene formations of southern Madagascar. Malagasy Nature.

[542] Tran LAP (2016). Interaction between Digestive Strategy and Niche Specialization Predicts Speciation Rates across Herbivorous Mammals. The American Naturalist.

[543] Treydte AC, Bernasconi SM, Kreuzer M, Edwards PJ (2006). Diet of the common warthog (*Phacochoerus africanus*) on former cattle grounds in a Tanzanian savanna. J. Mammal..

[544] Tsuji Y, Ito TY, Wada K, Watanabe K (2015). Spatial patterns in the diet of the Japanese macaque Macaca fuscata and their environmental determinants. Mammal Review.

[545] Tuboi C, Hussain SA (2016). Factors affecting forage selection by the endangered Eld’s deer and hog deer in the floating meadows of Barak-Chindwin basin of North-east India. Mammalian Biology.

[546] Turvey ST, Fritz SA (2011). The ghosts of mammals past: biological and geographical patterns of global mammalian extinction across the Holocene. Philos. Trans. R. Soc. Lond. B Biol. Sci..

[547] van Asperen EN, Kahlke R-D (2015). Dietary variation and overlap in Central and Northwest European Stephanorhinus kirchbergensis and S. hemitoechus (Rhinocerotidae, Mammalia) influenced by habitat diversity: “You’ll have to take pot luck!”(proverb). Quaternary Science Reviews.

[548] Van Den Bergh GD (2008). The youngest stegodon remains in Southeast Asia from the Late Pleistocene archaeological site Liang Bua, Flores, Indonesia. Quaternary International.

[549] van der Made, J. & Grube, R. in *Elefantentreich - Eine Fossilwelt in Europa* (eds D. Höhne & W. Schwarz) (Landesamt für Denkmalpflege und Archälogie Sachsen-Anhalt & Landesmuseum für Vorgeschichte, Halle, 2010).

[550] van der Made J, Tong HW (2008). Phylogeny of the giant deer with palmate brow tines Megaloceros from west and Sinomegaceros from east Eurasia. Quaternary International.

[551] van Dyck, S. & Strahan, R. *The Mammals of Australia*. (New Holland Publishing Australia Pty Ltd, 2008).

[552] Van Geel B (2018). Giant deer (Megaloceros giganteus) diet from Mid‐Weichselian deposits under the present North Sea inferred from molar‐embedded botanical remains. Journal of Quaternary Science.

[553] van Heteren AH, van Dierendonck RC, van Egmond MA, Sjang L, Kreuning J (2017). Neither slim nor fat: estimating the mass of the dodo (Raphus cucullatus, Aves, Columbiformes) based on the largest sample of dodo bones to date. PeerJ.

[554] Vandercone RP, Dinadh C, Wijethunga G, Ranawana K, Rasmussen DT (2012). Dietary diversity and food selection in Hanuman langurs (Semnopithecus entellus) and purple-faced langurs (Trachypithecus vetulus) in the Kaludiyapokuna Forest Reserve in the dry zone of Sri Lanka. Int. J. Primatol..

[555] Vasey, N., Burney, D. A. & Godfrey, L. R. in *Leaping Ahead* 149-156 (Springer, 2012).

[556] Velázquez NJ, Burry LS, Fugassa MH (2015). Palynological analysis of extinct herbivore dung from Patagonia, Argentina. Quaternary International.

[557] Venter JA, Kalule-Sabiti MJ (2016). Diet composition of the large herbivores in Mkambati nature reserve, eastern cape, South Africa. African Journal of Wildlife Research.

[558] Vizcaíno SF, Bargo MS, Cassini GH (2006). Dental occlusal surface area in relation to body mass, food habits and other biological features in fossil xenarthrans. Ameghiniana.

[559] Villarreal-Espino-Barros OA (2008). Composición botánica de la dieta del venado temazate rojo (Mazama temama), en la sierra nororiental del estado de Puebla. Universidad y ciencia.

[560] Vizcaíno SF, Bargo MS (1998). The masticatory apparatus of the armadillo Eutatus (Mammalia, Cingulata) and some allied genera: paleobiology and evolution. Paleobiology.

[561] Vizcaíno SF, Fariña RA, Fernicola JC (2009). Young Darwin and the ecology and extinction of Pleistocene South American fossil mammals. Revista de la Asociacion Geologica Argentina.

[562] Vizcaíno SF, Cassini GH, Fernicola JC, Bargo MS (2011). Evaluating habitats and feeding habits through ecomorphological features in Glyptodonts (Mammalia, Xenarthra). Ameghiniana.

[563] Vos, J. D. & Van de Geer, A. in *International Insular Investigations, V Deia International Conference of Prehistory* Vol. 1095 (eds Waldren & Ensenyat) 395–405 (2002).

[564] Wallach AD (2019). When all life counts in conservation. Conserv. Biol..

[565] Wang, B., Zhang, J. & Hu, J. Habitat selection by Chinese goral (Naemorhedus griseus) in spring in Fentongzhai Nature Reserve. *Sichuan Journal of Zoology* (2008).

[566] Wangchuk TR, Wegge P, Sangay T (2016). Habitat and diet of Bhutan takin Budorcas taxicolor whitei during summer in Jigme Dorji National Park, Bhutan. Journal of Natural History.

[567] Webb S (2008). Megafauna demography and late Quaternary climatic change in Australia: A predisposition to extinction. Boreas.

[568] Webb S (2009). Late Quaternary distribution and biogeography of the southern Lake Eyre basin (SLEB) megafauna, South Australia. Boreas.

[569] Wegge P, Shrestha AK, Moe SR (2006). Dry season diets of sympatric ungulates in lowland Nepal: competition and facilitation in alluvial tall grasslands. Ecol. Res..

[570] Weinberg PJ (2002). Capra cylindricornis. Mammalian Species.

[571] Werdelin, L. & Sanders, W. J. *Cenozoic mammals of Africa*. (University of California Press, 2010).

[572] White JL (1993). Indicators of locomotor habits in xenarthrans: evidence for locomotor heterogeneity among fossil sloths. Journal of Vertebrate Paleontology.

[573] White, T. G. & Alberico, M. S. Dinomys branickii. *Mammalian Species*, 1–5, 10.2307/3504284 (1992).

[574] Wikipedia. (2019).

[575] Williams KD, Petrides GA (1980). Browse use, feeding behavior, and management of the Malayan tapir. The Journal of Wildlife Management.

[576] Williams JB (1993). Field metabolism, water requirements, and foraging behavior of wild ostriches in the Namib. Ecology.

[577] Wilman H (2014). EltonTraits 1.0: Species-level foraging attributes of the world’s birds and mammals. Ecology.

[578] Wilson LAB, Sánchez-Villagra MR, Madden RH, Kay RF (2012). Testing a developmental model in the fossil record: molar proportions in South American ungulates. Paleobiology.

[579] Wingard G (2011). Argali food habits and dietary overlap with domestic livestock in Ikh Nart Nature Reserve, Mongolia. J. Arid Environ..

[580] Wood RJ (1992). The propagation and maintenance of the Arabian tahr Hemitragus jayakari at the Omani Mammal Breeding Centre, Bait al Barakah. The International Zoo Yearbook.

[581] Wood JR (2008). Coprolite deposits reveal the diet and ecology of the extinct New Zealand megaherbivore moa (Aves, Dinornithiformes). Quaternary Science Reviews.

[582] Wood JR, Richardson SJ, McGlone MS, Wilmshurst JM (2020). The diets of moa (Aves: Dinornithiformes). New Zealand Journal of Ecology.

[583] Woolnough AP, Steele VR (2001). The palaeoecology of the Vombatidae: did giant wombats burrow?. Mammal Review.

[584] Worthy T, Holdaway RN, Sorenson M, Cooper A (2009). Description of the first complete skeleton of the extinct New Zealand goose Cnemiornis calcitrans (Aves: Anatidae), and a reassessment of the relationships of Cnemiornis. J. Zool..

[585] Worthy T (1990). An analysis of the distribution and relative abundance of moa species (Aves: Dinornithiformes). New Zealand Journal of Zoology.

[586] Worthy T (2001). A giant flightless pigeon gen. et sp. nov. and a new species of Ducula (Aves: Columbidae), from Quaternary deposits in Fiji. Journal of the Royal Society of New Zealand.

[587] Worthy, T. H. & Holdaway, R. N. *The lost world of the moa: prehistoric life of New Zealand*. (Indiana University Press, 2002).

[588] Worthy TH (2016). Osteology supports a stem-galliform affinity for the giant extinct flightless bird Sylviornis neocaledoniae (Sylviornithidae, Galloanseres). PLoS One.

[589] Worthy TH, Degrange FJ, Handley WD, Lee MS (2017). The evolution of giant flightless birds and novel phylogenetic relationships for extinct fowl (Aves, Galloanseres). Royal Society open science.

[590] Wright, D. D. in *Tropical fruits and frugivores* 205-236 (Springer, 2005).

[591] Xiang ZF, Liang WB, Nie SG, Li M (2012). Diet and feeding behavior of Rhinopithecus brelichi at Yangaoping. Guizhou. Am. J. Primatol..

[592] Xu W (2012). Diet of Gazella subgutturosa (Güldenstaedt, 1780) and food overlap with domestic sheep in Xinjiang, China. Journal of Vertebrate Biology.

[593] Xu W (2012). Seasonal diet of khulan (Equidae) in northern Xinjiang, China. Italian Journal of Zoology.

[594] Yang Y (2019). First insights into the feeding habits of the Critically Endangered black snub-nosed monkey, Rhinopithecus strykeri (Colobinae, Primates). Primates.

[595] Yates AM, Worthy TH (2019). A diminutive species of emu (Casuariidae: Dromaiinae) from the late Miocene of the Northern Territory, Australia. Journal of Vertebrate Paleontology.

[596] YouTube. *YouTube*, www.youtube.com (Accessed May 2019).

[597] Zhang H, Wang Y, Janis CM, Goodall RH, Purnell MA (2017). An examination of feeding ecology in Pleistocene proboscideans from southern China (Sinomastodon, Stegodon, Elephas), by means of dental microwear texture analysis. Quaternary International.

[598] Zhegallo V (2005). On the fossil rhinoceros Elasmotherium (including the collections of the Russian Academy of Sciences). Cranium.

[599] Zheng, R. & Bao, Y. Seasonal food habits of the black muntjac Muntiacus crinifrons. *Europe PMC*, 201–207 (2010).

[600] Zhou Q, Wei H, Huang Z, Huang C (2011). Diet of the Assamese macaque Macaca assamensis in limestone habitats of Nonggang, China. Current Zoology.

[601] Zingg, A. *Seasonal variability in the diet composition of alpine ibex (Capra ibex ibex L.) in the Swis National Park* Masters thesis, University of Zurich (2009).

